# BPA Decreases PDCD4 in Bovine Granulosa Cells Independently of miR-21 Inhibition

**DOI:** 10.3390/ijms23158276

**Published:** 2022-07-27

**Authors:** Reem Sabry, Makenna Williams, Nicholas Werry, Jonathan LaMarre, Laura A. Favetta

**Affiliations:** Reproductive Health and Biotechnology Laboratory, Department of Biomedical Sciences, Ontario Veterinary College, University of Guelph, 50 Stone Rd. E, Guelph, ON N1G 2W1, Canada; rsabry@uoguelph.ca (R.S.); mwilli44@uoguelph.ca (M.W.); nwerry@uoguelph.ca (N.W.); jlamarre@uoguelph.ca (J.L.)

**Keywords:** miR-21, granulosa cells, PDCD4, PTEN, apoptosis, BPA

## Abstract

microRNAs (miRNAs) are susceptible to environmental factors that might affect cellular function and impose negative effects on female reproduction. miR-21 is the most abundant miRNA in bovine granulosa cells and is widely reported as affected by Bisphenol A (BPA) exposure, yet the cause and consequences are not entirely elucidated. BPA is a synthetic endocrine disruptor associated with poor fertility. miR-21 function in bovine granulosa cells is investigated utilizing locked nucleic acid (LNA) oligonucleotides to suppress miR-21. Before measuring apoptosis and quantifying miR-21 apoptotic targets PDCD4 and PTEN, transfection was optimized and validated. BPA was introduced to see how it affects miR-21 regulation and which BPA-mediated effects are influenced by miR-21. miR-21 knockdown and specificity against additional miRNAs were confirmed. miR-21 was found to have antiapoptotic effects, which could be explained by its effect on the proapoptotic target PDCD4, but not PTEN. Previous findings of miR-21 overexpression were validated using BPA treatments, and the temporal influence of BPA on miR-21 levels was addressed. Finally, BPA effects on upstream regulators, such as VMP1 and STAT3, explain the BPA-dependent upregulation of miR-21 expression. Overall, this research enhances our understanding of miR-21 function in granulosa cells and the mechanisms of BPA-induced reproductive impairment.

## 1. Introduction

The epigenetic landscape of gene regulation is a well-known phenomenon with increasing advancements in biotechnology over the last several decades [1]. Epigenetic mechanisms are underlying players in every biological process in all bodily systems including nervous, cardiovascular, endocrine, and reproductive systems [2]. Epigenetic mechanisms include DNA methylation, histone modifications, and regulatory non-coding RNAs [1]. The latter involves the function of various non-coding RNAs that were considered “junk” RNA when first discovered, but are now emerging as crucial gene expression modulators [3]. microRNAs (miRNAs) are types of non-coding RNAs that regulate gene expression post-transcriptionally by binding to complementary messenger RNAs (mRNAs) and either degrading the transcript or inhibiting translation into a functional protein [4].

All epigenetics mechanisms are vulnerable to environmental influences that ultimately alter cellular function. This can have undesirable consequences on all biological systems, but there is a particular concern regarding disruptions to the female reproductive system [5]. Precise miRNA regulation within oocytes and granulosa cells (GCs) is of utmost importance as the molecular environment within these cell types is rapidly changing to a greater extent than any other cell in the female body [5]. Any disruptions in the maternal epigenome can be trans-generationally inherited by the embryo and offspring, reducing its survival potential [6]. Therefore, investigations into environmental influences on miRNA regulation are warranted to advance our understanding of mechanisms ultimately regulating oocyte competency and female fertility. Most of the transcriptional activity is turned off during oocyte maturation and the oocyte is considered quiescent [7]. The required nutrients and molecules are accumulated in the oocyte or are transported into oocytes from neighboring GCs [8]. GCs are necessary for oocyte competence and are often used to infer the developmental capabilities of oocytes and early embryos. Oocytes communicate with GCs which produce small molecules that get shuttled back into the oocyte [9]. miRNAs travel from GCs to the oocyte through gap junctions [10], making it imperative to explore miRNAs in granulosa cells to understand their role in oocyte maturation and embryo development. 

miR-21 has been repeatedly reported as one of the most abundant miRNAs in oocytes and granulosa cells of various mammalian species, including murine, ovine, bovine, porcine, and humans [11,12,13,14]. miR-21 expression significantly increases in oocytes during maturation [12,15] and one suggested explanation is the transport of miR-21 from the GCs into the oocyte [15]. This miRNA has multiple important functions within bovine and human GCs including, but not limited to, cell cycle progression, proliferation, differentiation, migration, and inflammation, and is largely acknowledged as an inhibitor of apoptosis and an oncomiR implicated in cancers [10,16,17]. Two main targets of miR-21 are programmed cell death 4 (PDCD4) and phosphatase tensin homolog (PTEN), which induce apoptosis and repress tumor progression, respectively [18]. One target will damage cells while the other protects them; this depicts the complex nature of miR-21’s function and the importance of maintaining miR-21 at optimal levels. In fact, reduced miR-21 levels result in increased apoptosis [16], while increased miR-21 is damaging and carcinogenic to follicular cells [19,20]. 

The primary form of miR-21 (pri-miR-21) is an intronic gene that is found within the 10th coding intron for vacuole membrane protein 1 (VMP1); therefore, each time VMP1 is transcribed, miR-21 should theoretically be coexpressed [7]. However, miR-21 possesses its own upstream promotor than can activate independently of VMP1 expression [7]. One transcription factor, STAT3, is known to bind to these promoters in bovine granulosa cells and is an activator of pri-miR-21 transcription through the miR-21 promoter [15]. Hence, VMP1 and STAT3 are upstream regulators of miR-21 at the transcriptional level. Furthermore, pri-miR-21 transcription can also be regulated by estrogen signaling as miR-21 promotors can be controlled by an upstream estrogen response element (ERE) [21]. Regulation by estrogens renders miR-21 transcription susceptible to endocrine-disrupting compounds (EDCs) [21].

The most common widespread EDC is Bisphenol A (BPA), a plasticizer used in manufacturing, which can mimic estrogen and inappropriately induce a cellular response [22]. BPA is found in food containers, thermal receipts, pipelining, cleaning supplies, cosmetics, and surgical equipment [22] as well as in the environment (water, soil, and air) [21] and in most bodily tissues of invertebrates (zebrafish) and vertebrates (rodents, equine, bovine, ovine, porcine, and humans) [23]. It has been detected in oocytes, granulosa cells, and embryos with negative correlations to oocyte competence and embryo development. In bovine oocytes, granulosa cells, and early embryos, BPA has been shown to induce oxidative stress, alter sex ratios, disrupt gap junctions, and extensively dysregulate gene expression [24,25,26,27]. BPA has been shown to affect epigenetics, such as DNA methylation and histone modification, in part, by disrupting the activity of methyltransferase and acetylase enzymes [8,28,29]. BPA alters miRNA expression profiles [30,31,32,33,34] with multiple reports of BPA-dependent increases in miR-21 expression in bovine oocytes and granulosa cells [27], in breast cancer cells [35], in human ovarian cancer cells [36], in human placental cells [37,38], and in mice pancreatic cells [39]. In a previous study from our group, BPA not only increased the expression of mature miR-21 but also pri-miR-21 in bovine COCs [27]. This suggests that BPA-mediated increases in miR-21 expression occur at the transcriptional level, but the exact cause is not elucidated, and the consequences of miR-21 disruption are not yet characterized. 

Research into the mechanistic regulation and functional roles of miR-21 in granulosa cells is lacking in the bovine species, although it is crucial as cattle are invaluable livestock whose reproductive success provides food security and economic welfare. In the following study, we investigate miR-21 function in bovine granulosa cells by inhibiting miR-21 using locked nucleic acid (LNA) oligonucleotides complementary to miR-21. Transfection was optimized and validated before measurement of apoptosis and quantification of miR-21 targets PDCD4 and PTEN. Moreover, this study also examines BPAs effects on miR-21 and determines which BPA-mediated effects may be regulated by miR-21. Altogether, these data provide insight into the function of miR-21, and support further use of this model for investigating BPA toxicity in female reproduction.

## 2. Results

### 2.1. miR-21 Expression Increases 12 h after BPA Treatment in Bovine Granulosa Cells

**Figure 1:**
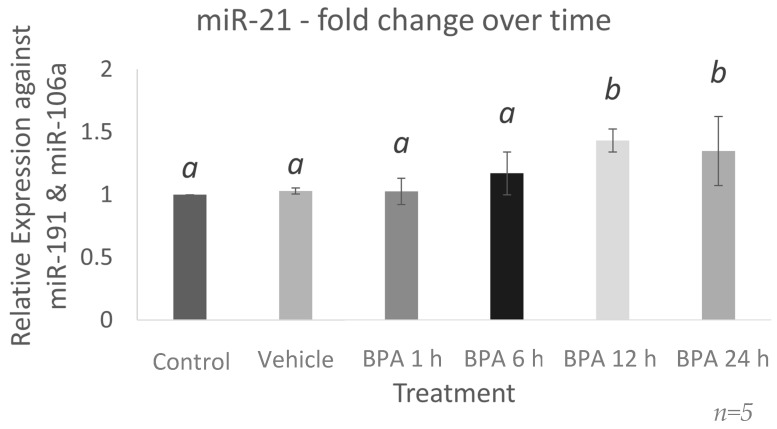
**mi-21-fold change over time after BPA treatment**. miR-21 significantly increased after 12 and 24 h of BPA treatment. All samples originated from in vitro cultured granulosa cells treated with 0.05 mg/mL BPA for 1, 6, 12, and 24 h. Quantification is relative to reference targets miR-191 and miR-106a. Different letters indicate significant differences, with *b* indicating a significantly different mean than *a* at *p* < 0.05. Bars represent the mean ± SEM.

To determine the duration of exposure necessary for BPA to alter miR-21 levels, qPCR was used to measure miR-21 in granulosa cells 1, 6, 12, and 24 h after BPA treatment. As shown in Figure 1, BPA significantly increased miR-21 expression at 12 h (*p* = 0.0412) with a 1.4-fold increase in miR-21 expression from 1 h to 12 h. This effect remained significant after 24 h (*p* = 0.0464). Further experiments with BPA treatments were conducted at 12 h exposure periods.

Additionally, the effect of in vitro granulosa cell culture on miR-21 expression was evaluated. Supplemental Figure S1 shows miR-21 expression increases 5-fold upon plating at P0 (*p* = 0.0001) then decreased from P0 to P2 (*p* = 0.033). Serum was then removed after 24 h of P2 culture, and no significant effect was observed. The serum component was eliminated as a variable factor: transfections and BPA treatments were performed on granulosa cells that had been starved with OptiMEM at 24 h after plating.

### 2.2. Optimal Uptake Efficiencies of miR-21 and Scramble LNAs Occur after 12 h of Transfection at 0.5 μM Concentrations

**Figure 2:**
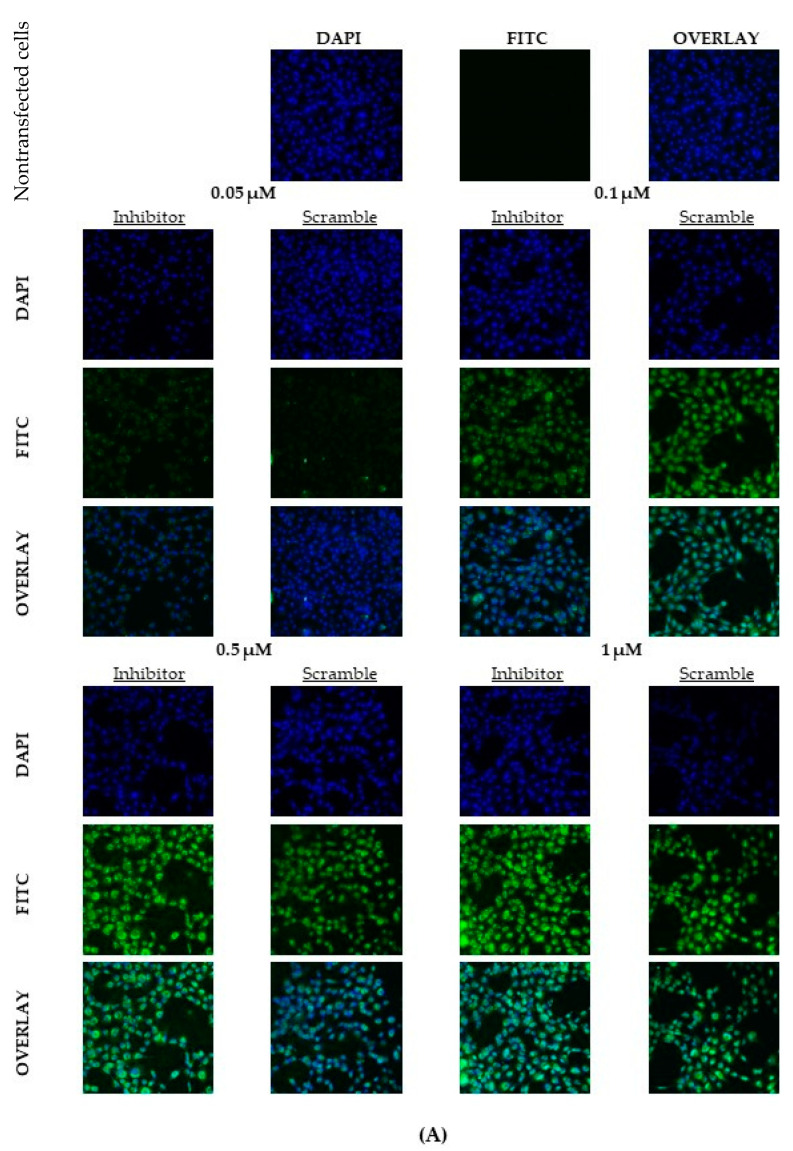

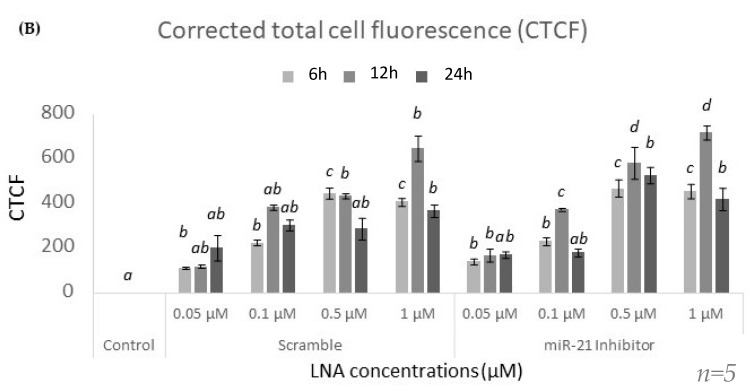
**Confocal microscopy of LNAs at different concentrations and quantification of fluorescence intensities**. Transfections with FAM-labeled LNA probes at 0.05 μM, 0.1 μM, 0.5 μM, and 1 μM for 6, 12, and 24 h. (**A**) depicts a representation of confocal images taken at 12 h post transfection. In vitro cultured granulosa cells were transfected, fixed on slides, counterstained with Hoescht stain, and imaged on an Olympus FV1200 Confocal Microscope. Quantification was done through the ImageJ software; the CTCF (**B**) showed significantly increased transfection efficiencies at the higher concentrations (0.5 μM and 1 μM). Different letters indicate significant differences, with *b* indicating a significantly different mean than *a* at *p* < 0.001 and *c* indicating a significantly different mean than *a* and *b* at *p* < 0.05. *ab* indicates no differences between *a* or *b*. Bars represent the mean ± SEM.

Transfection efficiencies were calculated using FAM-labelled LNA probes for both the miR-21 inhibitor and the scramble control. Initially, fluorescence confocal microscopy was used to visualize the uptake into the cells and fluorescence intensity analysis using ImageJ was used to quantify the transfection efficiencies of our probes at 4 different LNA concentrations (0.05 μM, 0.1 μM, 0.5 μM, and 1 μM). Figure 2A depicts the representative time point of 12 h with the fluorescence intensities of the varying concentrations. The confocal images for the additional time points tested (6 and 24 h) can be found in Supplementary Figure S2A,B. It is evident from visual observations, the higher LNA concentrations of 0.5 μM and 1 μM are more efficient at transfecting the cells with higher FITC staining in these groups corresponding to increased FAM signaling from the LNA probes.

Supplementary Figure S2C represents the number of cells that exhibited a green, fluorescent signal. At all three time points, 0.05 μM of both LNAs was not sufficient to transfect the granulosa cells with an average number of cells with a green signal lying below 20%. All other concentrations resulted in over 80% of cells with a green signal. It is important to note that this value represents the number of green cells and does not consider the varying intensities of the FAM signal that is directly proportional to the transfection efficiencies. To account for this, Figure 2B displays the corrected total cell fluorescence (CTCF) for each group. The CTCF shows that optimal fluorescence occurs at the two highest concentrations of 0.5 μM and 1 μM for both LNAs tested (*p* < 0.05). For both the inhibitor and the scramble, peak fluorescence intensity occurred at 12 h and declined after 24 h, this decline was significant for the inhibitor but not the scramble. Through confocal microscopy and ImageJ analysis, the higher concentrations of 0.5 μM and 1 μM after 12 h of transfection present the most promising conditions for optimal efficiencies.

**Figure 3:**
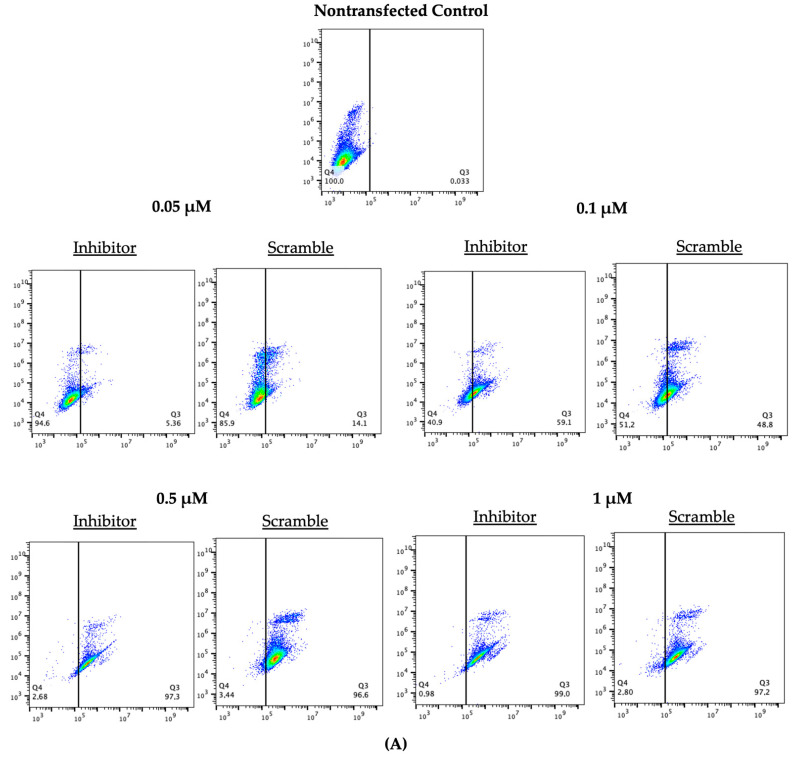

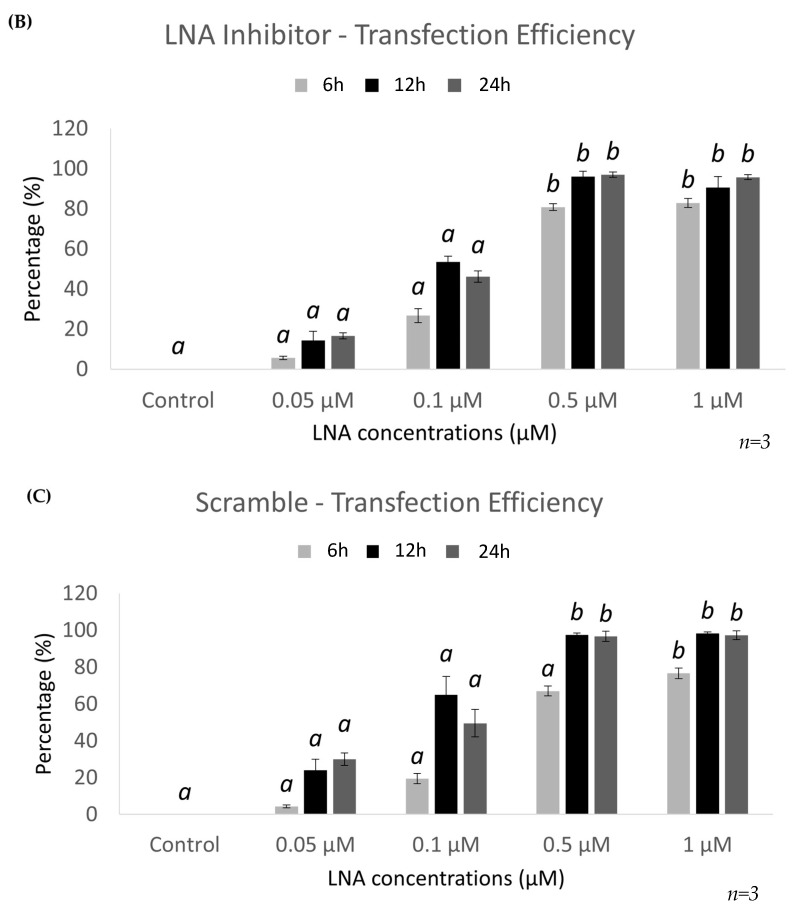
**Flow Cytometry of LNAs at different concentrations**. Transfections with FAM-labeled LNA probes at 0.05 μM, 0.1 μM, 0.5 μM, and 1 μM for 6, 12, and 24 h. (**A**) depicts a representation of flow cytometry scatter plots measured at 12 h post transfection. In vitro cultured granulosa cells were transfected, trypsinized, counterstained with Hoescht stain, and run through a BD Accuri C6 Flow Cytometer. Transfection efficiencies of the inhibitor (**B**) and the scramble (**C**) showed significantly increased transfection efficiencies at the higher concentrations (0.5 μM and1 μM). Different letters indicate significant differences, with *b* indicating a significantly different mean than *a* at *p* < 0.05. Bars represent the mean ± SEM.

Flow cytometry was used to confirm transfection efficiencies. As shown in the 12 h representative scatter plots in Figure 3A, green, fluorescent signals were found in more than 90% of cells at concentrations of 0.5 μM and higher for both LNAs tested at 12 h. This is apparent in the gradual shifts of the flow scatter plots in Figure 3A. The plots move from the left negative quadrants to the right positive transfection quadrants as the concentration increases. Significantly increased transfection efficiencies for the inhibitor LNA were found at 6, 12, and 24 h for both 0.5 μM and 1 μM concentrations (*p* < 0.05) and the scramble efficiencies were significantly increased at 12 and 24 h for 0.5 μM concentrations and at 6, 12, and 24 h for 1 μM concentrations (*p* < 0.05) (Figure 3B,C). The scatter plots for the additional time points tested (6 and 24 h) can be found in Supplementary Figure S3. Overall, the transfection efficiencies quantified through flow cytometry confirm those calculated through ImageJ; signifying that 0.5 μM and 1 μM at 12 and 24 h exhibit the most efficient transfections.

### 2.3. Knockdown of miR-21 Confirmed by qPCR Leads to Functional Differences on the Protein Level but Not at the Transcript Level of Known miR-21 Targets

**Figure 4:**
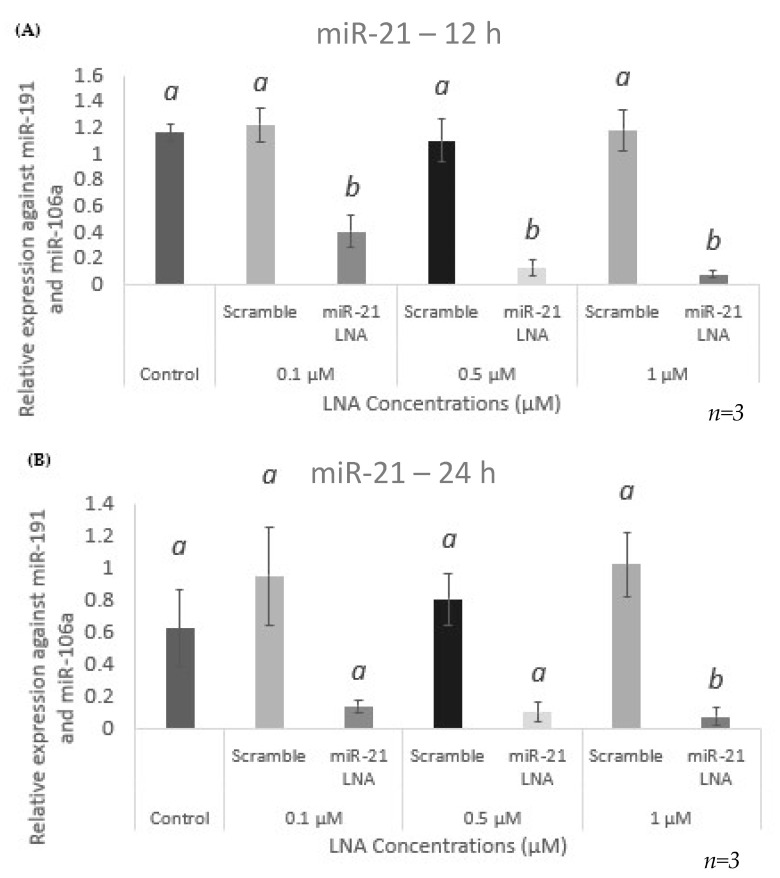
**mi-21 expression at different LNA concentrations**. miR-21 was significantly decreased in all three concentrations tested at 12 h but the difference was only significant for 1 μM at 24 h. Transfections with LNA probes at 0.1 μM, 0.5 μM, and 1 μM for 12 h (**A**) and 24 h (**B**). Quantification is relative to reference targets miR-191 and miR-106a. Different letters indicate significant differences, with *b* indicating a significantly different mean than *a* at *p* < 0.001. Bars represent the mean ± SEM.

miR-21 expression in nontransfected cells was quantified using qPCR and compared to levels of miR-21 in cells transfected with the scramble and the miR-21 inhibitor LNAs. Taking into consideration the low transfection efficiencies seen at both the 0.05 μM concentration and at 6 h of transfection time, these were eliminated from further analysis. Results show that miR-21 expression was significantly reduced in the miR-21 LNA treated groups at 12 h for all three concentrations tested (Figure 4A) with a higher significance at 0.5 μM and 1 μM (*p* < 0.0001) than at 0.1 μM (*p* = 0.0008). At 24 h, although miR-21 was expressed in low amounts in all concentrations tested, this reduced expression was only significant at 1 μM of the miR-21 LNA (*p* = 0.05) (Figure 4B). 

**Figure 5:**
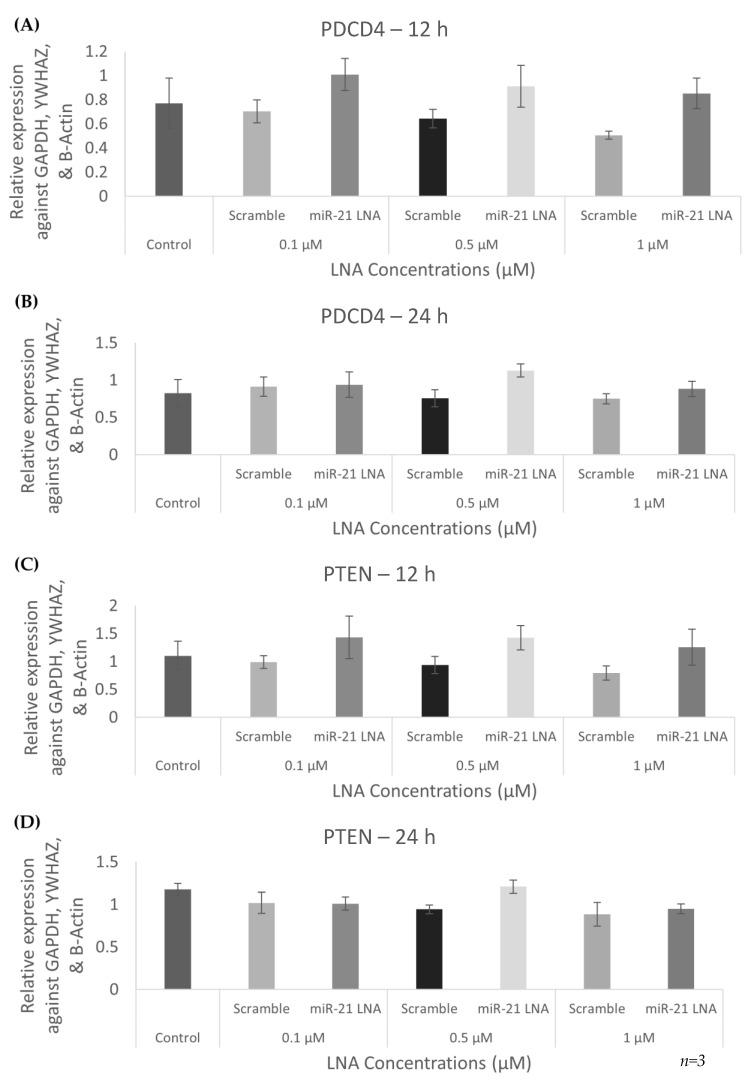
**Expression of mRNA targets at different LNA concentrations**. PDCD4 (**A**,**B**) and PTEN (**C**,**D**) transcripts were unaffected by miR-21 LNA transfections. Cells were transfected with LNA probes at 0.1 μM, 0.5 μM, and 1 μM for 12 h (**A**,**C**) and 24 h (**B**,**D**). Quantification is relative to reference genes YWHAZ and b-actin. Bars represent the mean ± SEM.

Quantification of known miR-21 targets, PDCD4 and PTEN, at both the transcript and protein level is used to confirm or reject a functional knockdown of miR-21 in this study. Looking at the transcript levels at three concentrations tested at both 12 and 24 h of transfection revealed no significant changes in mRNA expression of targets after miR-21 LNA treatment (Figure 5A–D). To determine the functional consequences of miR-21 LNA treatment, it is crucial to quantify proteins.

**Figure 6:**
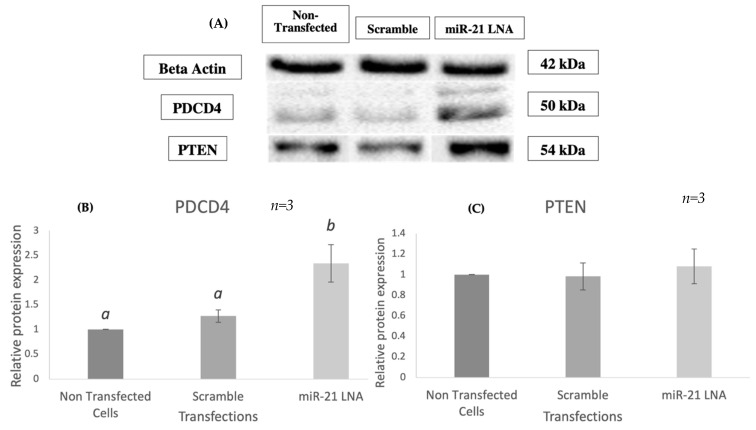
**Relative protein expression of PDCD4 & PTEN after miR-21 inhibition**. Western blots (**A**) and graphical representations of PDCD4 (**B**) and PTEN (**C**) revealed that miR-21 inhibition significantly increased PDCD4 protein levels but did not affect PTEN. Transfections were done with LNA inhibitor probes at 0.5 μM for 12 h. Densitometric analysis was performed relative to the loading control, β-actin. PTEN representation contains lane rearrangement; full original PTEN blot is included in the Supplementary File (Figure S4). Different letters indicate significant differences, with *b* indicating a significantly different mean than *a* at *p* < 0.005. Bars represent the mean ± SEM.

From transfection efficiencies and qPCR expression levels data, the optimal concentrations for miR-21 inhibition are 0.5 μM and 1 μM, while the optimal transfection times are 12 or 24 h. The cytotoxic nature of LNAs strongly encourages the utilization of the lowest concentration and transfection time possible [40]: 0.5 μM of both LNAs for 12 h. Western blotting revealed that miR-21 LNA treatment significantly increased the expression of the PDCD4 protein levels (*p* = 0.0058) (Figure 6A,B), while no changes were observed in PTEN protein levels (Figure 6C) when quantified against the loading control B-actin. PDCD4 increased expression strengthens the knockdown validation.

### 2.4. Specificity of miR-21 LNAs Shown through No Effects on Other miRNAs except for the Significantly Increased Expression of miR-155

**Figure 7:**
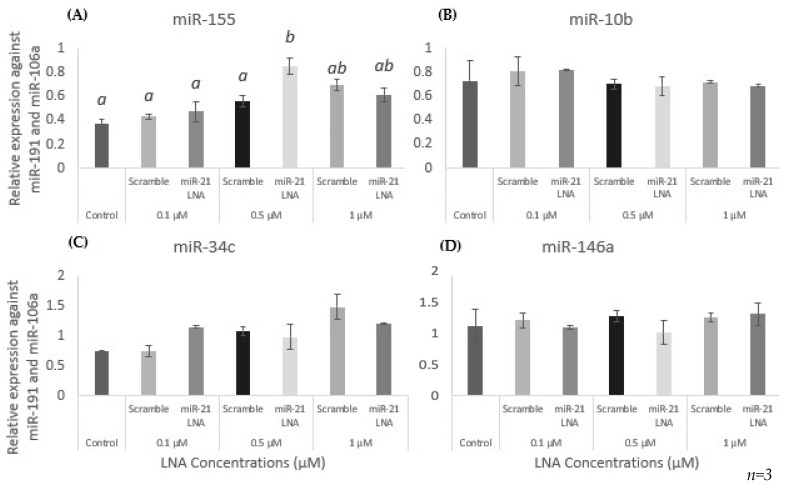
**Expression of other miRNAs at different LNA concentrations**. miR-155 (**A**) was the only miRNA with induced expression after miR-21 LNA treatment at 0.5 μM. All other miRNAs: miR-10b (**B**), miR-34c (**C**), and miR-146a (**D**) were unaffected. Transfections with LNA probes at 0.1 μM, 0.5 μM, and 1 μM for 12 h. Quantification is relative to reference targets miR-191 and miR-106a. Different letters indicate significant differences, with *b* indicating a significantly different mean than *a* at *p* < 0.001. *ab* indicates no differences between *a* or *b*. Bars represent the mean ± SEM.

In addition to miR-21 quantification, other miRNAs highly expressed in granulosa cells were quantified to ensure the specificity of the miR-21 LNA. qPCR results are shown in Figure 7A–D with no measurable effect on other miRNAs (miR-155, 10b, 34c, and 146a) except for an induction of miR-155 at 0.5 μM (Figure 7A) (*p* = 0.0005). 

### 2.5. Knockdown of miR-21 Induces Apoptosis in Granulosa Cells through Positive Annexin Staining

**Figure 8:**
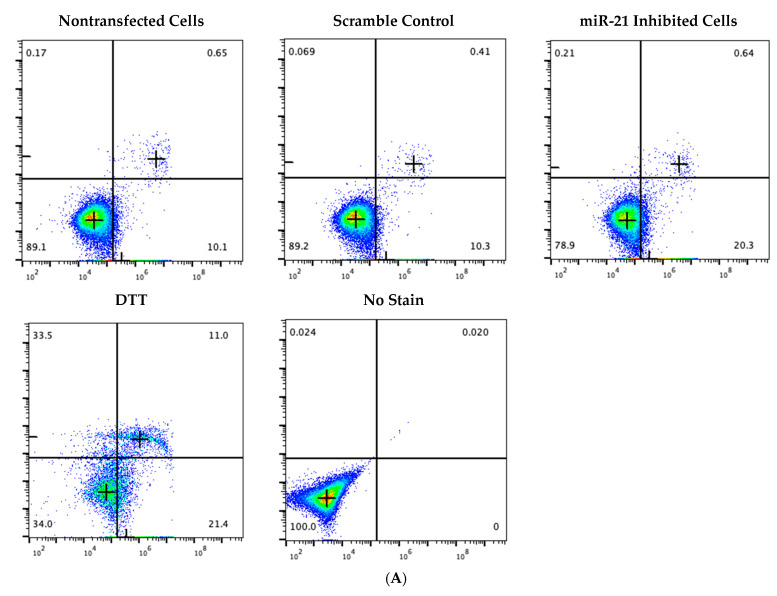

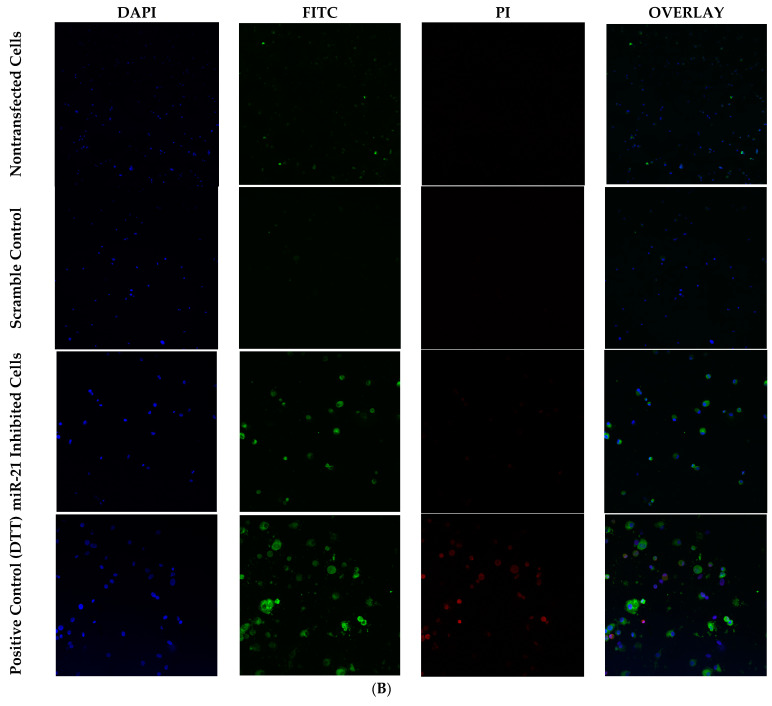

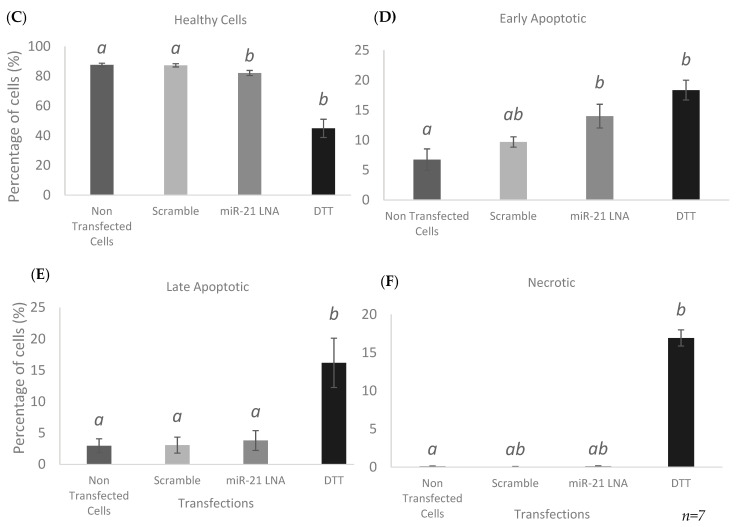
**Annexin V/PI staining for apoptosis detection after miR-21 LNA treatment**. Transfected cells were stained with Annexin V and PI to measure apoptosis using flow cytometry. Flow scatter plots (**A**), fluorescent images (**B**), and FlowJo quantification results (**C**–**F**) show that miR-21 inhibition decreases and increases the percentage of healthy cells (**C**) and early apoptotic cells (**D**), respectively, like the positive control, DTT. Different letters indicate significant differences, with *b* indicating a significantly different mean than *a* at *p* < 0.05. *ab* indicates no differences between *a* or *b*. Bars represent the mean ± SEM.

The binding of Annexin V to phosphatidylserine present on apoptotic cells enables an annexin V assay to adequately detect and quantify levels of apoptosis within cell populations among different treatment groups. PI staining allows for the detection of late apoptotic or necrotic cells. Figure 8A displays the scatter plots obtained from flow cytometry. As represented in the bottom right quadrants, cells treated with miR-21 LNAs exhibited a 2-fold increase in early apoptotic cells like the positive control tested, DTT. This is supported in Figure 8B where remaining cells from flow readings were placed on slides and imaged under confocal microscopy. The images clearly depict increased FITC staining in the miR-21 inhibited cells like the positive control when compared to the nontransfected cells and the scramble control cells.

The quantitative and statistical analysis shown in Figure 8C–F display graphical representations of the diagrams. miR-21 inhibition significantly decreases and increases healthy and early apoptotic cells, respectively. A significant decrease in healthy cells (Figure 8C) was found in both miR-21 inhibited cells (*p* = 0.0395) and DTT (*p* = 0.001) as well as significant increases in early apoptotic cells (Figure 8D) for both miR-21 LNA (*p* = 0.0203) and DTT (*p* = 0.0048) treated cells. There was no statistical difference between early apoptotic cells in the scramble and inhibitor groups (Figure 8D). It is apparent that miR-21 inhibition under these conditions induces early apoptosis in bovine granulosa cells with no changes in the percentage of late apoptotic and necrotic cells (Figure 8E,F). Only the positive control, DTT, significantly increased the percentage of late apoptotic (*p* = 0.0181) and necrotic (*p* = 0.0461) cells.

### 2.6. BPA Affects miR-10b in Addition to miR-21, but LNA-21 Treatment Affects miR-155

**Figure 9:**
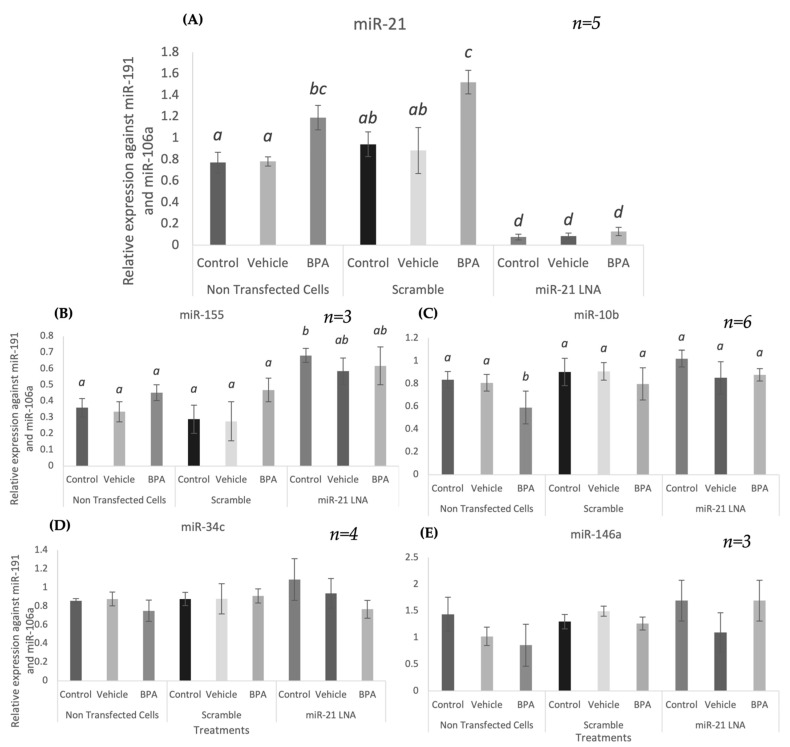
**Expression of miRNAs after miR-21 inhibition and BPA treatment**. Cells were transfected with LNA probes at 0.5 μM for 12 h then treated with BPA (0.05 mg/mL) for another 12 h. BPA and miR-21 LNAs continue to increase and decrease miR-21 expression, respectively (**A**). BPA decreased miR-10b (**B**) and miR-155 (**C**) remained upregulated after miR-21 LNA treatment at 0.5 μM. All other miRNAs: miR-34c (**D**), and miR-146a (**E**) were unaffected by both treatments. Quantification is relative to reference targets miR-191 and miR-106a. Different letters indicate significant differences, with *b* indicating a significantly different mean than *a* at *p* < 0.05, *c* indicating a significantly different mean than *a* and *b*, and *d* indicating a significantly different mean than *a*, *b*, and *c* at *p* < 0.05. *ab* indicates no differences between *a* or *b* and *bc* indicates no differences between *b* or *c*. Bars represent the mean ± SEM.

Transfected cells were treated with BPA at the current reported LOAEL dose of 0.05 mg/mL [24,27]. In addition to BPA, cells were also treated with an ethanol vehicle in which BPA is dissolved. As previously observed, miR-21 was significantly increased after BPA treatment in the nontransfected group (*p* = 0.0185) and in the scramble group (*p* = 0.0299) (Figure 9A). Once again, miR-21 expression was significantly decreased in the miR-21 LNA treated groups (*p* < 0.05). This decreased expression masks any effects of BPA on miR-21 resulting in no changes between the control, vehicle, and BPA treated groups within the miR-21 LNA treated groups.

Investigations into the effects of BPA and miR-21 inhibition on the other miRNAs previously tested revealed that BPA significantly decreased miR-10b expression (*p* = 0.0339) within the nontransfected group; however, this was not observed in both LNA treated groups (Figure 9C). As previously stated, miR-155 was significantly increased in the miR-21 LNA treated control group (*p* = 0.0049) (Figure 9B). Lastly, neither miR-21 inhibition nor BPA treatment affected miR-34c and 146a expression (Figure 9D,E).

### 2.7. BPA Increases the Expression of the miR-21 Transcription Factor, STAT3, and the Primary Form of miR-21, VMP1-miR-21

**Figure 10:**
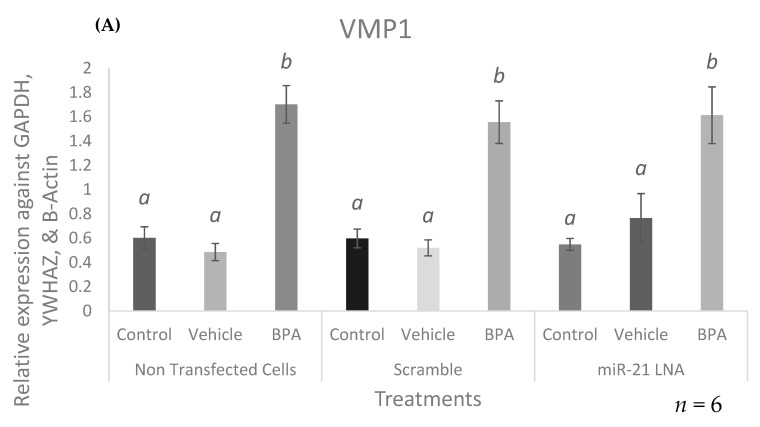

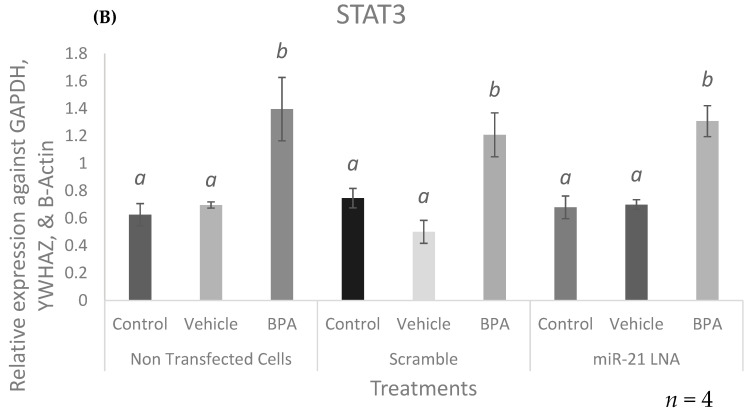
**mRNA expression of modulators upstream of miR-21 transcription**. Cells were transfected with LNA probes at 0.5 μM for 12 h then treated with BPA for another 12 h. Upstream miR-21 regulators, VMP1 (**A**), and STAT3 (**B**) were upregulated after BPA treatment. Quantification is relative to reference genes GAPDH, YWHAZ, and B-Actin. Different letters indicate significant differences, with *b* indicating a significantly different mean than *a* at *p* < 0.05. Bars represent the mean ± SEM.

To determine the potential causes of BPA-mediated increase in miR-21, two crucial genes that modulate miR-21 transcription upstream of mature miR-21 expression were quantified by qPCR. VMP1, a long transcript that contains the primary code of miR-21, was found to be significantly increased after BPA treatment in all three cell types: in the nontransfected cells (*p* = 0.0051), the scramble treated cells (*p* = 0.0051), and in the miR-21 LNA treated cells (*p* = 0.0074) as shown in Figure 10A. STAT3, a crucial transcription factor that induces miR-21 transcription, was also found to be significantly increased after BPA exposure: in the nontransfected cells (*p* = 0.0386), the scramble treated cells (*p* = 0.05), and in the miR-21 LNA treated cells (*p* = 0.0449) as shown in Figure 10B.

### 2.8. Known miR-21 Target Transcripts and Proteins Were Affected by BPA, but These Changes Were Not Reversed or Exaggerated by LNA-21 Knockdown

**Figure 11:**
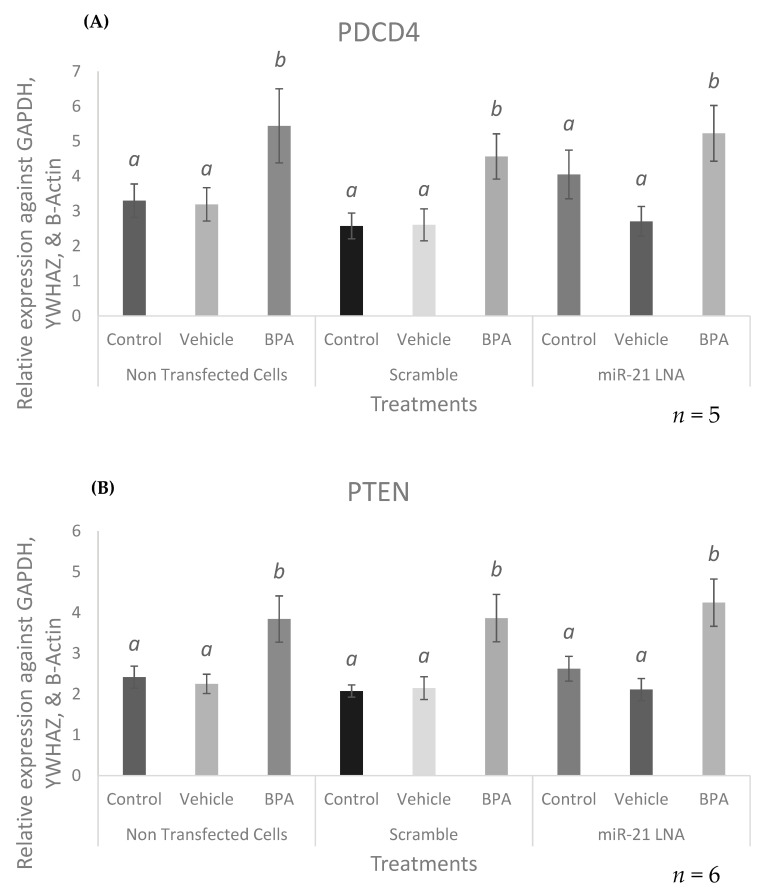
**Target mRNA expression of PDCD4 and PTEN after miR-21 LNA and BPA treatment**. Cells were transfected with LNA probes at 0.5 μM for 12 h then treated with BPA for another 12 h. PDCD4 (**A**), and PTEN (**B**) were significantly increased after BPA treatment. Quantification is relative to reference genes GAPDH, YWHAZ, and B-Actin. Different letters indicate significant differences, with *b* indicating a significantly different mean than *a* at *p* < 0.05. Bars represent the mean ± SEM.

Analysis of PDCD4 and PTEN in miR-21 LNA + BPA treated cells is crucial to begin to understand the apoptotic pathway utilized by BPA in granulosa cells. Initially, the transcript levels of these genes were quantified in the presence of either an LNA or BPA or both. PDCD4 and PTEN mRNA expression was significantly increased after BPA exposure in all three cell types. PDCD4 transcripts were increased in the nontransfected cells (*p* = 0.0279), the scramble treated cells (*p* = 0.0483), and in the miR-21 LNA treated cells (*p* = 0.0205) as shown in Figure 11A. PTEN transcripts were also increased in the nontransfected cells (*p* = 0.0325), the scramble treated cells (*p* = 0.0468), and in the miR-21 LNA treated cells (*p* = 0.0066) as shown in Figure 11B.

**Figure 12:**
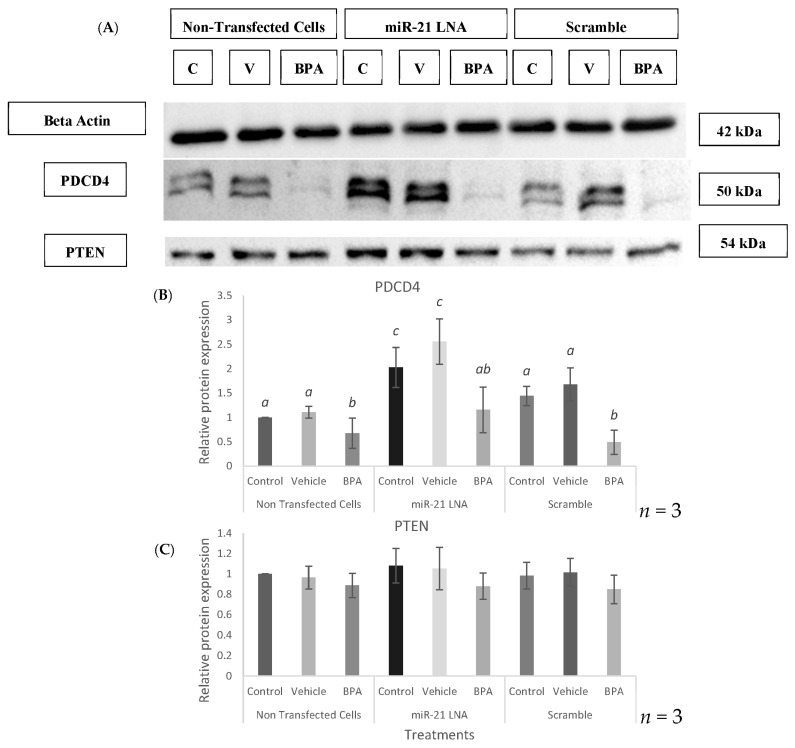
**Relative protein expression of PDCD4 & PTEN after miR-21 inhibition and BPA treatment**. Western blots (**A**) and graphical representations of PDCD4 (**B**) and PTEN (**C**) revealed that BPA decreased PDCD4 protein, but miR-21 LNA treatment increased it. Neither BPA nor miR-21 LNA treatment affected PTEN protein. Transfections were done with LNA inhibitor probes at 0.5 μM for 12 h followed by BPA treatment for another 12 h. Densitometric analysis was performed relative to the loading control, B-actin. Different letters indicate significant differences, with *b* indicating a significantly different mean than *a* at *p* < 0.05 and *c* indicating a significantly different mean than *a* and *b* at *p* < 0.05. *ab* indicates no differences between *a* or *b*. Bars represent the mean ± SEM.

At the protein level, Western blot analysis revealed that BPA significantly reduced PDCD4 protein levels in the nontransfected cells (*p* = 0.0043), the scramble treated cells (*p* = 0.0496), and in the miR-21 LNA treated cells (*p* = 0.023) as shown in the graph and blot in Figure 12A,B. Interestingly, the levels of PDCD4 in the BPA treated miR-21 inhibited group is similar to the levels of PDCD4 in the nontransfected and scramble controls, suggesting a small but clear recovery in BPA mediated decreases in PDCD4 protein. Both BPA and miR-21 LNA treatment did not have any effect on the protein levels of PTEN (Figure 12C).

## 3. Discussion

The oocyte and its surrounding granulosa cells can be influenced by the follicle’s distinct molecular microenvironment. miRNAs are crucial players that can regulate thousands of genes and conversely, a single gene could be inhibited through a variety of miRNAs [22]. The miRNA expression profile in granulosa cells varies depending on the stage of development [5]. GC miRNAs work together to govern meiotic progression via regulating proliferation, steroidogenesis, and apoptosis, all of which help to promote oocyte maturation [8]. They have also been linked to disorders, such as infertility, PCOS, EDC exposure, and numerous malignancies [10,19,27]. BPA, a widespread EDC, is ubiquitous in the environment and in bodily tissues with endless routes of exposure [21]. Unmetabolized BPA can readily pass-through phospholipid bilayers and accumulate within cells and it has been shown to be a reproductive toxicant [8]. The elaborate process of miRNA biosynthesis involves a network of components and enzymes that can be disrupted by EDCs, like BPA [41]. 

There is a plethora of mechanisms BPA can utilize to alter miRNA functions: methylation levels of miRNA promoters [42], activity of miRNA activators/inhibitors, miRNA transcription factors [43]. Studies have reported BPA-induced disruptions of cell cycle progression, apoptosis, and steroidogenesis [44,45,46], all of which are largely regulated by miRNAs [31]. It is warranted to investigate the role of miRNAs in the presence/absence of BPA to better understand the mechanisms that regulate miRNA expression and the potential downstream consequences of toxicants’ exposure. 

miR-21 is one of the most abundant miRNAs in female reproductive tissues [12,15] and is overexpressed in oocytes and granulosa cells of patients with reduced fertility, PCOS, and ovarian cancers [20,47,48]. Although its abundance is essential for normal physiological functions, its presence in excess may have deleterious effects. Previous studies from our group showed miR-21 overexpression after BPA exposure in bovine granulosa cells, with increased expression of the primary form [27]. However, the origin and aftermath of this effect were not yet explicated. 

In this study, miR-21 expression was reduced in in vitro cultured bovine granulosa cells using locked nucleic acid (LNA) inhibitors to characterize the functional role of miR-21 and to examine the outcome of these cells following BPA exposure. This study showed that miR-21 exhibits antiapoptotic properties that could be explained by its influence on the proapoptotic target, PDCD4, but not PTEN. Investigating the effects of BPA on upstream regulators, VMP1 and STAT3, provided a plausible explanation for BPA-induced miR-21 expression. miR-21 baseline expression significantly increased upon plating and serum withdrawal did not influence miR-21 expression when compared to passage 2 cells grown entirely in serum-containing media. The increase in miR-21 upon plating can be explained by the removal of GCs from their native in vivo environments surrounding the oocyte to an alternative in vitro culture environment. In vitro cell culture is known to induce stress to cells and activate several factors involved in cell signaling and survival [49]. miR-21, being an antiapoptotic regulator, is considered a survival factor and could explain the increase observed [19].

miR-21 expression was reduced using LNAs to characterize its role in GCs and to investigate its role in apoptosis. This study also optimized and validated LNA transfection conditions for our specific model to achieve an efficient and functional knockdown of miR-21. LNAs contained a backbone modification that allows them to be less sensitive to nuclease activity and have a higher rate of uptake into cells without the use of a transfection reagent, which can exhibit confounding toxic effects on the cells [50,51]. For unassisted uptake, the manufacturer recommended a higher concentration of the LNAs ranging from 0.1–1 μM. In an effort to minimize cytotoxic effects and confounding results, the least amount of exogenous oligonucleotides possible while maintaining optimal knockdowns should be used [51] with 0.5 μM of LNAs being the ideal concentration for transfections in our experiments. It is preferred to inhibit miR-21 for a shorter period as it is a crucial factor for cell survival [51,52]; therefore, 0.5 μM of miR-21 LNA and scramble LNA for 12 h were used as there is evidence to support adequate transfection under these conditions, while simultaneously mitigating potentially undesirable effects of this cellular manipulation.

miR-21 expression levels were successfully and significantly reduced after miR-21 LNA treatment at all three concentrations at both 12 h and 24 h. LNA specificity was confirmed as there were no significant reductions of any other miRNA (miR-155, -10b, -34c, -146a) tested. Interestingly, miR-155 did display significantly increased expression in the group transfected with 0.5 μM of miR-21 LNA, hinting at a phenomenon that is not well understood: miRNA to miRNA interaction [53]. This occurs when miRNAs self-regulate other miRNAs and influence their expression [53]. For example, miR-483 is a self-regulating miRNA that indirectly upregulates its own activating transcription factor, USF1, which subsequently enhances its own transcription [54]. Hill & Tran [55] extensively discuss known miRNA-miRNA interactions and their potential roles in various cancers, and recent work has identified miRNA-miRNA correlations associated with male fertility [56]. Although there is no documented interaction between miR-21 and miR-155, our findings suggest that miR-21 is capable of regulating miR-155 through direct or indirect interactions. Research in this field is new and emerging: regulation of miRNAs abundance, biosynthesis, and ultimately function on mRNA modulation can be attained via another miRNA [53]. 

The reduced expression of miR-21 alone in our LNA treated groups is supported by another study that knocked down miR-21 with LNAs in mice granulosa cells and found its expression to be one twenty-seventh of that in GCs treated with the scramble, which was similar to the nontransfected cells [52]. This confirms that miR-21 is expressed in extremely low amounts in our cells but does not tell us whether miR-21 was functionally silenced. To do this, both mRNA and protein levels of well-known miR-21 targets, PDCD4 and PTEN, were quantified in our knockdown model. This can help to determine if miR-21s interaction with these targets results in mRNA degradation or simply inhibition of protein translation. At the mRNA level, miR-21 inhibition did not alter PDCD4 or PTEN. miR-21 inhibition significantly increases PDCD4 protein, but not PTEN. The increased PDCD4 protein not only validates a functional knockdown in this study, but also supports the antiapoptotic function of miR-21 [30]. PDCD4 is a proapoptotic factor that possesses a 3′UTR seed sequence that is strongly complementary to miR-21 [12]. Therefore, part of miR-21’s antiapoptotic functions is to bind to and repress PDCD4 apoptotic actions [12]. Inhibition of miR-21 is expected to increase PDCD4 expression and is likely to induce apoptosis. This is in agreement with several studies in the literature that show increased PDCD4 expression and increased apoptosis in miR-21 inhibited models [1,12].

When apoptosis was quantified using an annexin V assay, GCs treated with the miR-21 LNA resulted in significantly decreased and increased percentages of healthy and early apoptotic cells, respectively. Reduced levels of miR-21 occur simultaneously with increased levels of PDCD4 protein. PDCD4 can induce apoptosis through its role in the AKT pathway [57]. miR-21 inhibition in endometrial cells increased PDCD4 expression which inhibited AKT phosphorylation; without active AKT, cell proliferation was repressed, and migration of endometrial cells was inhibited [57]. Another study describes PDCD4 involvement in the JNK signaling pathway with their pro-apoptotic activity [58]. Green et al. [59] also demonstrate that PPARy receptors can inhibit miR-21 expression, thereby enhancing PDCD4 in hypoxic smooth muscle cells. This resulted in inhibition of proliferation and induction of apoptosis [59]. The miR-21-PDCD4 axis of apoptotic regulation is a well-recognized pathway widely documented, further strengthening the findings in this study. 

On the other hand, PTEN was not changed at the RNA or protein levels after miR-21 inhibition. This unexpected result is still reliable when considering the complex nature of miRNA regulation: one gene can be regulated by hundreds of miRNAs [22]. In fact, PTEN is found to be tightly regulated by other miRNAs, including miR-19 and miR-494 [60,61]. In miR-21 absence, PTEN can still be regulated by other factors that could explain the lack of changes observed. Other studies have reported a direct cause and effect on miR-21 and PTEN in other species and cell types [62,63,64]. This indicates that miR-21 regulation of its different targets could be species- and cell-specific, adding another layer of complexity to miRNA function. In our model, miR-21 must repress PDCD4 to a greater degree than PTEN. 

Transfected cells were then also treated with BPA at 0.05 mg/mL. Numerous reports show that BPA can alter specific miRNAs influencing their overall functions [31,33,65]. We previously showed BPA-induced upregulation of miR-21 in bovine oocytes and GCs [27] yet the cause and effect of these changes are not well characterized. The time for BPA to significantly increase miR-21 was determined as a baseline effect, expression levels were quantified in GCs after BPA exposure at 1, 6, 12, and 24 h. Although several studies have described elevated miR-21 expression after BPA exposure [35,36,38,39], to the best of our knowledge, this is the first study to determine the onset of such effects in GCs. BPA had no effect on miR-21 after 1 and 6 h of exposure with significantly increased expression at 12 and 24 h with the highest expression at 12 h, chosen as BPA treatment time. The time it takes for BPA to alter miRNA expression depends on several factors including the rate of BPA uptake into the cells, the concentration tested, the species and tissue/cell types, and individual differences in BPA metabolism. This increase persisted until 24 h of exposure, mimicking the effects that would be observed in GCs over the course of oocyte maturation as this process takes 24 h to happen in the bovine species [66]. High levels of miR-21 reduced GC expansion, decreased meiotic progression, inhibited antioxidants expression, and altered gene expression [10]. Carcinogenic effects of BPA can be supported by its ability to disrupt miRNAs expression. Another study similarly found oncomiR-21 to be overexpressed after BPA exposure which was correlated with BPA-induced uncontrolled proliferation of MCF-7 cells [35]. Considering BPA has been found to exhibit the exact same effects in GCs, suggests an important link between BPA and miR-21 regulation that ultimately impacts normal oocyte and GC function.

miR-21 and other miRNAs were also quantified in our transfected/treated models to confirm the specificity of the miR-21 LNA and to validate previous findings regarding BPA-mediated alteration on miRNA expression. As previously mentioned, miR-21 was significantly increased and decreased after BPA treatment and miR-21 LNA treatment, respectively. The effects of BPA on miR-21 were apparent in the scramble and nontransfected groups; however, the knockdown of miR-21 masks BPA effect, a possible foundation for therapeutic treatments to counteract bisphenol toxicity. miR-155 was the only other miRNA affected by miR-21 inhibition but was not increased after BPA exposure as previously described [27]. However, the previous study treated GCs for 24 h while this study exposed GCs to BPA for 12 h, suggesting that BPA-mediated increases in miR-155 expression require an exposure period longer than 12 h. Similar to the previous study, miR-10b was significantly reduced after BPA exposure in nontransfected cells, while miR-34c and -146a were unaffected [27]. 

BPA is an estrogen mimic linking it to a variety of estrogen-mediated pathways [21]. miR-21 is regulated by an estrogen element upstream of its promoter, rendering it susceptible to BPA influence [67]. Therefore, a probable mechanism of action is the binding of BPA to nuclear estrogen receptors (ER), which then dimerize and translocate into the nucleus where they bind to EREs upstream of miR-21 [68]. This interaction can then activate miR-21 transcription resulting in elevated miR-21 levels. Even though this pathway explains the observed effects, it is unlikely that this is the sole mechanism responsible since BPA increased miR-21 in ER-deficient cell lines [35]. Therefore, there must be multiple underlying mechanisms of BPA that cooperate to induce miR-21 expression.

Despite having its own promoter, miR-21 might be co-expressed with its parent gene, VMP1, in which it resides [69]. To test VMP1 involvement in BPA-induced miR-21 expression, VMP1 was quantified after BPA exposure, showing a significantly increase. This supports that BPA-induced VMP1 expression contributes, in part, to increased co-expression of its 10th intronic gene, pri-miR-21. To the best of our knowledge, this is the first study to report the effects of BPA on the VMP1 gene. Despite its connection to miR-21, VMP1, a gene required for organelle biogenesis, is described as a stress-induced protein that is also found to be highly expressed in cancerous cells [70,71] and to lead to vacuole formation and apoptosis [72]. As BPA is proapoptotic, a BPA-dependent VMP1 increase fits its role. On the other hand, no changes in VMP1 expression after noticeable changes in miR-21 are also reported [52]. It is plausible that other factors and pathways could influence miR-21 independently of VMP1 expression. 

The activating transcription factor, STAT3, is another regulator potentially influencing miR-21 upregulation after BPA treatment [15] as BPA significantly increased STAT3 expression in our study. A study by Tscherner et al. [15] showed that the promoter region of miR-21 contains two binding sites for STAT3 in bovine granulosa cells and when bovine COCs were treated with a STAT3 inhibitor, pri-miR-21 significantly decreased. Similar to our study, Zhang et al. [43] also found STAT3 expression increased after BPA exposure in MCF-7 cells. High levels of STAT3 will undoubtedly result in increased binding to the miR-21 promoter and increased activation of transcription. STAT3 possesses an array of functions including proliferation, differentiation, angiogenesis, and apoptosis [73]. Like miR-21, aberrantly high levels of STAT3 in various malignancies are correlated with poor cancer prognosis [74]. Zhang et al. [43] concluded that BPA-induced STAT3 activation contributes to induced proliferation in breast cancer cells. Interestingly, these effects were reversed once they blocked the actions of epidermal growth factor receptors (EGFR), suggesting that BPA’s association with breast cancer is dependent on STAT3 signaling [43]. This raises the possibility that BPA’s mechanism of action in certain cancers is dependent on STAT3/miR-21 signaling and regulation, which together contribute to poor prognosis and outcomes.

The downstream apoptotic targets of miR-21, PDCD4 and PTEN, were quantified at the transcript and protein levels in this transfected/treated model to evaluate the effects of BPA on these molecules and whether miR-21 inhibition mitigates any of these effects. At the RNA level, BPA significantly increased the expression of both PDCD4 and PTEN with no changes amongst nontransfected cells, scramble cells, and miR-21 LNA treated cells. Interestingly, at the protein level PDCD4 showed an inverse effect to their mRNA counterparts, with a significant decrease in protein after BPA exposure. It is speculated that the increase in mRNA expression is due to a translational block of the protein. It is plausible that BPA initially interferes with the translation of PDCD4 thereby decreasing protein levels. If this is a crucial protein required by the granulosa cells, they can compensate for this reduction in protein by offsetting the levels of transcripts present in the cell [75]. In fact, the levels of mRNA to protein in a cell is not always directly proportional and can be influenced by several factors such as turnover/stability of the transcripts and proteins [75] as well as numerous mechanisms that can act on genes post transcriptionally [75]. This is further supported by our study which looks at the involvement of a microRNA, which also acts through post transcriptional regulation. If BPA increases miR-21, then it is likely binding to PDCD4 transcripts and preventing their translation without necessarily degrading them. This will explain the accumulation of PDCD4 mRNA with significant decreases in PDCD4 protein.

In miR-21 absence, BPA still reduces PDCD4, however, levels in the miR-21 transfected and BPA treated group is not significantly different than the levels found in the nontransfected/scramble controls, suggesting minor recovery of PDCD4 protein. It is speculated that BPA induces an antiapoptotic and prooncogenic effect through miR-21 and PDCD4 regulation. This is supported by the observation of BPA induced increases in antiapoptotic miR-21 with simultaneous decreases in proapoptotic PDCD4. The decrease in PDCD4 protein after miR-21 inhibition implies the presence of a second pathway by which BPA decreases PDCD4 that is possibly compensating for a miR-21 knockdown. Therefore, it is imperative to mention that BPA might still be affecting PDCD4 through miR-21, yet these effects are masked behind an alternative pathway that is independent of miR-21 expression. It is also likely that this alternate pathway is not as efficient as the miR-21 pathway, thereby, explaining the minor recovery of PDCD4 protein observed in the inhibited group. The levels of PDCD4 were slightly increased and comparable to the nontransfected and scramble controls.

One potential pathway compensating for a reduction in miR-21 is the involvement of another miRNA, including miR-499 [76]. This is the most likely pathway for several reasons: it is well known that microRNAs have numerous targets and that all targets can be regulated by several miRNAs in order to compensate for the disruption of a singular pathway [22]. In addition to this, Ajuyah et al. [76] describes how miR-21 is the main contributor in PDCD4 regulation but upon further investigation found that several miRNAs are weaker regulators that can bind to PDCD4 mRNA. They explored miR-499 for its involvement in PDCD4 regulation in various cancers [77]. Lastly, a recent study has also documented that BPA treatment of pregnant rats significantly increased miR-499 expression in their hearts [17]. Altogether, these findings support the speculation that although miR-21 is the predominant regulator of PDCD4 protein, there are alternate pathways that could explain BPA mediated decreases in PDCD4 independent of miR-21. BPA could similarly increase miR-499 in these granulosa cells, which could compensate for low miR-21 and continue to inhibit PDCD4 to produce oncogenic effects. The notion that miR-21 is a stronger regulator of PDCD4 than miR-499 could explain the minor recovery of PDCD4 protein seen in this study.

A decrease in a proapoptotic compound is not necessarily positive. Cells exposed to BPA display several deleterious effects including oxidative stress, DNA fragmentation, and countless other disruptions [25,26]. If these damaged unhealthy cells are prevented from undergoing apoptosis and allowed to persist and potentially replicate, this could lead to the activation of cancerous processes within the cell [78,79]. In fact, one of the most general concerns with BPA is its carcinogenic tendencies: it is detected at a higher level in most cancers and, as previously stated, so is miR-21 [35,36]. BPA has been shown to inhibit the expression of several apoptotic genes, such as caspases, while inducing in ovarian cancers the expression of pro-survival genes, such as Bcl-x1, which promotes tumor growth [80]. Dumitrascu et al. [80] suggest that the BPA model closely mimics molecular conditions of several pathologies including PCOS, poor fertility, and various cancer types that are potentially independent of estrogen signaling. This is supported by BPA-induced increases and decreases of miR-21 and PDCD4, respectively, as well as a partial recovery of PDCD4 in the BPA treated miR-21 inhibited cells. Perhaps, utilizing a combination of miR-21 inhibition and PDCD4 supplementation targeted at cancerous cells with high BPA exposure could be sufficient in inducing apoptosis to these damaged cells and reducing the risks of uncontrolled proliferation associated with malignancies. Teng et al. [81] reported that an alternative EDC, Fenhexamide, stimulated miR-21 expression and reduced PDCD4 protein which contributed to the survival of malignant breast cancer cells [81].

Another unexpected observation is the lack of changes to PTEN protein levels after BPA exposure. A study in Sertoli cells found that BPA increased PTEN expression which contributed to increased apoptosis [82]. Another study found contradictory results with decreased PTEN expression after BPA exposure in rat mammary glands [83]. This latter study also reported that when BPA was correlated with a soy component (Genistein), there was no effect on PTEN expression [83]. Although this does not directly explain our results, it does suggest a potential mechanism by which PTEN expression is protected from the actions of BPA; PTEN may not be a direct target of both BPA and miR-21 in bovine granulosa cells. It is important to note that miR-21 is not only involved in apoptosis, but has been functionally linked to the establishment of differentiation or pluripotency in bovine embryos [84], production of aromatase and estradiol in granulosa cells [85], and inhibition of tumor suppressor genes [10,30]. The disruptions in miR-21 expression via BPA can influence each of these processes in a unique manner that will ultimately contribute to poor fertility and increased risk of reproductive disorders.

## 4. Materials and Methods

### 4.1. Granulosa Cells Collection and In Vitro Granulosa Cell Culture

Bovine ovaries (Bos taurus) were obtained from a local abattoir (Highland Packers, Stoney Creek, ON, Canada). Ovaries were transported at a temperature between 34–36 °C and cumulus-oocyte complexes (COCs) were aspirated from follicles and collected into collection media comprised of 1 M HEPES-buffered Ham’s F-10 media (Sigma-Aldrich, St. Louis, MO, USA) supplemented with 2% steer serum (Cansera, Etobicoke, ON, Canada), Heparin (2 IU/mL) (Sigma-Aldrich), Sodium Bicarbonate (ThermoFisher, Whitby, ON, Canada), and Penicillin/Streptomycin (1%) (Gibco, Whitby, ON, Canada). Approximately 100 COCs were collected and denuded of their granulosa cells using mechanical disruptions via a micropipette. Granulosa cells were washed in phosphate-buffered saline (PBS) (Wisent, Saint-Jeane Baptiste, QC, Canada) and in 1× Dulbecco’s Modified Eagle Medium (DMEM) (Gibco), glutamine (2 mM) (Sigma-Aldrich), and penicillin/streptomycin (1%). Cells were resuspended in DMEM supplemented with 20% fetal bovine serum (FBS) (Wisent) and cultured in T25 flasks at 38.5 °C in 5% CO_2_ for 6–7 days with media replacement every 48 h. At 100% confluency, the cells were passaged twice until passage 2 (P2) for all subsequent experiments.

To determine the baseline effects of in vitro granulosa cell culture on miR-21 expression, fresh granulosa cells were collected and compared to cells at the end of passage 0 (Po), passage 1 (P1), and passage 2, P2. To mimic the culture conditions used during transfection, another group of P2 cells was cultured in serum for 24 h then serum restricted in an OptiMEM media (Gibco) prior to collection. To determine the time at which BPA induces miR-21 expression, P2 granulosa cells were grown in serum for 24 h in 6 well plates then serum restricted for another 24 h before being treated with BPA (0.05 mg/mL) (Sigma Aldrich) for 1, 6, 12, and 24 h. This was also used to determine the time of BPA exposure after transfections. In both cases, cells were trypsinized (0.25% Trypsin) (Wisent), collected, and frozen in liquid nitrogen for subsequent RNA analysis of miR-21.

### 4.2. Cell Transfections with Locked Nucleic Acids (LNAs)

The anti-miR-21 Locked Nucleic Acid (LNA) and the nonspecific scrambled control LNA were purchased from Qiagen (Toronto, ON, Canada). The LNA sequence was complementary to miR-21: 5′-CAACATCAGTCTGATAAGCT-3′, and the scrambled control was a random mix of nucleotides: 5′-TAACACGTCTATACGCCCA-3′. To allow for fluorescent visualization of incorporation into granulosa cells and for quantification of transfection efficiencies LNAs were synthesized with a fluorescein dye, 6-carboxyfluorescein (6-FAM) label modification on the 5′ end. After optimization, LNAs with no modifications were used and prepared according to manufacturers’ instructions.

P2 granulosa cells were split into 9 different groups in DMEM containing serum (10% FBS) in 6 well plates at 1 × 10^5^ cells/mL. After 24 h, cells were serum restricted using OptiMEM for another 24 h before being transfected with the LNAs. Time- and dose-LNA dependent experiments were performed to determine optimal dose and time for transfection. Cells were transfected with 0.05 μM, 0.1 μM, 0.5 μM, or 1 μM of both LNAs including a nontransfected control for 6, 12, and 24 h to determine the optimal time for transfection with limited cell death for downstream analysis. After transfections, cells were imaged using confocal microscopy (Olympus FV1200 microscope; Olympus Lifescience Solutions, Tokyo, Japan), processed using flow cytometry (BD Accuri C6 Flow Cytometer; BD Biosciences, Ann Arbor, MI, USA), or frozen (stored at −80°C) for downstream RNA or protein quantification by qPCR and Western blotting, respectively. Once the transfection conditions were determined, transfected cells were treated with a vehicle (0.1% ethanol) or BPA (0.05 mg/mL in 0.1% ethanol) in OptiMEM for 12 h then frozen in liquid nitrogen and stored at −80 °C for RNA and protein analysis. This dose of BPA is the currently reported LOAEL dose, which was previously determined to be a physiologically and environmentally relevant dose in bovine reproduction [24,27]. 

### 4.3. Confocal Microscopy

Confocal microscopy was used to visualize LNA uptake into granulosa cells, using ImageJ. Cells were grown as previously described but on coverslips. After treatments, coverslips were washed in PBS and cells were fixed using 4% paraformaldehyde (PFA) (FisherScientific) and kept in the dark for 1 h at room temperature. Cells were then counterstained with Hoechst Stain 33258 (ThermoFisher) for 5 min before being mounted onto slides precleaned in 80% methanol (FisherScientific, Portsmouth, NH, USA) with DakoCytomation Fluorescent Mounting Medium (Agilent Technologies, Santa Clara, CA, USA). Slides were then imaged using an Olympus FV1200 confocal microscope (Olympus, Tokyo, Japan) under two channels to detect the FAM-labeled LNAs and the nuclear Hoechst stain. Cells were imaged at a 20× objective, and 5 different fields of view were captured containing 100–150 cells/view on three biological replicates. Data shown represent the average fluorescence of three replicates where each replicate represents the average fluorescence of 500 cells per group. 

Images were analyzed using ImageJ software, and the number of cells fluorescing green was divided by the total number of cells per view (fluorescing blue) to obtain the percentage of cells with a positive LNA signal. Fluorescence intensity was measured using the formula for corrected total cell fluorescence (CTCF) [86]:

[Integrated Density − (Area of selected cell × Mean fluorescence of background readings)]

### 4.4. Flow Cytometry

Flow cytometry was used to further confirm transfection efficiency with cells grown and transfected as previously described, but without coverslips. Cells were dissociated from the well after transfections using StemPro Accutase (Gibco), as it is crucial for the cells to remain alive for subsequent flow readings and trypsin can have negative side effects after prolonged exposure [87]. After PBS washes and centrifugation at 5 rpm for 10 min at 6 °C, cells were resuspended in PBS containing Hoescht stain to account for the total cell population. The cell suspension was then strained through 40 μM strainers (VWR, Radnor, PA, USA) into flow SIP tubes (FisherScientific) to break apart any clumps of cells. Cells were run through the SIP and 50,000 events were counted for each group using the BD C6 Accuri Flow Cytometer (BD Biosciences, Mississauga, ON, Canada) with excitation/emission wavelengths of 488/533 and 488/670 for the FAM label and Hoechst stain, respectively. The data obtained were analyzed using the FlowJo^TM^ v10.8 software where the cell populations were gated based on the nontransfected control group; any populations outside the boundary of the quadrant were considered positively transfected with the LNAs. Three biological replicates were analyzed.

### 4.5. RNA Isolation and cDNA Synthesis

Total RNA was isolated using the miRNeasy Micro Kit (Qiagen, Toronto, ON, Canada) according to the manufacturer’s instructions from three biological replicates. RNA concentration and quality were measured using the Nanodrop 2000c (ThermoFisher). 1 μg of mRNAs and miRNAs were reverse transcribed (RT) using qScript complementary DNA (cDNA) Supermix (Quantabio, Beverly Hills, MA, USA) and qScript microRNA cDNA Synthesis kit (Quantabio), respectively, in a T100 Thermal Cycler (BioRad, Mississauga, ON, Canada). cDNA was diluted with RNase-free water to a concentration of 5 ng/μL (mRNA) and 1.5 ng/μL (miRNA) for qPCR amplification.

### 4.6. Quantitative qPCR

mRNA and miRNAs expression levels of a minimum of three biological replicates were quantified via quantitative real-time PCR (qPCR) using a CFX96 Touch Real-Time PCR Detection System (BioRad). mRNA was amplified using the SsoFast EvaGreen Supermix (BioRad) while miRNAs were amplified using the PerfeCTa SYBR Green Supermix (Quantabio). All primers were purchased from Sigma-Aldrich and were tested using standard curves with efficiencies accepted only with values between 90–110%. Gene expression was calculated using the efficiency-corrected method (ΔΔCt). Primer sequences and efficiencies can be found in Table 1 and Table 2. mRNA expression was normalized to housekeeping genes *Glyceraldehyde 3-phosphate dehydrogenase (GAPDH)*, *Tyrosine 3-monooxygenase/tryptophan 5-monooxygenase activation protein zeta (YWHAZ)*, and *Beta-actin (ACTB)*, as they were determined to be the most stable reference genes according to a GeNorm Analysis (Figure S5) using the CFX Maestro Software 2.3. miRNA expression was normalized to miR-191 and miR-106a, as they are stable reference targets across treatments [27]. All quantification was run on at least three biological replicates in technical triplicates.

miRNA PCR signal acquisition was carried out using the following three step PCR cycling protocol: 95 °C for 2 min followed by 39 cycles of 95 °C for 5 s, 60 °C for 30 s, 70 °C for 30 s, ending with melt curve acquisition from 60–95 °C. mRNA PCR signal acquisition was carried out using the following two step PCR cycling protocol: 95 °C for 2 min followed by 44 cycles of 95 °C for 10 s, 60 °C for 30 s, ending with melt curve acquisition from 60–95 °C.

### 4.7. Apoptosis Measurement Using Annexin Staining and Flow Cytometry

For the detection of apoptosis within cells transfected with the LNAs, an Annexin V Kit (ab14085—Abcam, Cambridge, UK) was used following the manufacturer’s instructions. Briefly, cells were grown as previously described and transfected with the dose and time of 0.05 μM for 12 h. Overall, there were 7 groups for this experiment: Nontransfected control, Scrambles control, miR-21 inhibited cells, positive control for apoptosis (Dithiothreitol—DTT) (Sigma-Aldrich), and three controls required for flow compensation (No stain, one group stained with annexin-FITC only, and one group stained with propidium iodide—PI only). 

Both culture media containing free-floating dead cells and adherent cells were analyzed. Pelleted cells were resuspended in 250 μL Binding Buffer containing 1% FBS and incubated at room temp for 5 min. 10 μL of Annexin-V FITC was added (except for the no stain and the PI only groups) and incubated in the dark for 30 min at room temperature. 5 μL of PI was added (except for the no stain and the Annexin only groups) and incubated for 5 min at room temp. The cells were then strained through 40 μM strainers (VWR) and immediately analyzed by flow cytometry, reading 50,000 events per group with excitation/emission wavelengths of 488/533 and 488/670 for Annexin-FITC and PI, respectively.

This kit can distinguish between live healthy cells, early apoptotic cells, late apoptotic cells, and necrotic cells using cell membrane integrity as the measurable endpoint. Live healthy cells possess a specific molecule in the inner leaflet of the phospholipid bilayer known as phosphatidyl serine [99]. The annexin-FITC antibody is specific to this molecule and is unable to bind when it is internalized in healthy cells. PI is a stain that is readily taken up by damaged cells with poor membrane integrity. Therefore, cells that exclude both FITC and PI stains are considered healthy cells. Cells that begin to undergo apoptosis will externalize phosphatidyl serine to the outer leaflet of the membrane, thereby exposing it to Annexin-FITC [99]. During early stages of apoptosis, this molecule will translocate before the breakdown of the cell membrane [99]. Therefore, cells that stain positive for FITC but negative for PI are considered early apoptotic cells. Once the membrane disintegrates, cells will stain positive for both FITC and PI and are then considered late apoptotic cells. Finally, dead necrotic cells can no longer maintain phosphatidyl serine expression and the positive FITC signal will fade while PI will remain positive. Therefore, cells that exclude FITC but stain positive for PI are considered necrotic.

The data were analyzed by FlowJo where the overlap between the two channels was compensated based on the stain controls, the granulosa cell populations were gated and a minimum of 5000 cells/events were included in analysis, and the apoptotic/necrotic populations were sectioned into quadrants based on the no stain control. Populations lying to the left of the x-axis are considered apoptotic, and those lying on the top half of the y-axis are considered necrotic. At least three biological replicates were measured.

### 4.8. Protein Isolation and Western Blot Analysis

All Western blotting buffers and reagents were made in-house unless otherwise specified. 

Quantification of PDCD4 and PTEN protein was performed by Western blotting on a minimum of 3 biological replicates. Samples were lysed in 50 μL radioimmunoprecipitation assay (RIPA) buffer and 1% (*v*/*v*) protease inhibitors (Biotool, Jupiter, FL, USA), followed by freeze–thaw cycles in liquid nitrogen. Samples were then sheared using 0.33 mm (29 G) syringes (BD Biosciences) to break down the clumping of genomic DNA, placed in a water bath sonicator for 30 min followed by centrifugation at 12,000× *g* RPM at 4 °C for 10 min. Protein concentrations were quantified using the Bio-Rad DC protein assay (BioRad) and 30 and 20 μg of proteins for PDCD4 and PTEN, respectively, were loaded onto gels. Equal volumes of 3× reducing buffer with β-mercaptoethanol (Sigma-Aldrich) were added to each sample. Polyacrylamide gels (12%) were prepared using Bio-Rad standard gel recipes. 

Proteins were heated for denaturation, separated on the 12% gels in an Invitrogen wet transfer Western blot apparatus (Invitrogen, Burlington, ON, Canada) at 125 V for 2 h and then transferred (35 V for 2 h) onto nitrocellulose membranes (Biorad) using a transfer buffer of Tris, Glycine, and water. Nitrocellulose blots were washed in Tris-buffered saline pH 7.6 with 0.1% Tween 20 (Thermo Fisher Scientific, Whitby, ON, Canada) (TBST), blocked for 1 h in 5% Bovine Serum Albumin (BSA) (Sigma-Aldrich) in TBST to limit nonspecific binding and incubated with each target primary antibody at 4 °C overnight: PDCD4 at 1:1000 (ThermoFisher; PA528150) and PTEN at 1:750 (Cell Signaling, Whitby, ON, Canada; 138G6) dilutions, respectively.

After TBST washes, blots were incubated with the anti-rabbit IgG HRP-linked secondary antibody (Cell signaling Technology; 70,745) at 1:3000 dilution for 1 h at room temp and incubated with Clarity Western ECL Blotting Substrate (Bio-Rad) for 3 mins. Blots were imaged on a ChemiDoc XRS  +  Imaging System (Bio-Rad). β-Actin (Cell Signalling Technology; 4967) was used as a loading control and densitometric analysis was performed using the Bio-Rad Image Lab software and quantified as a ratio to β-actin expression.

### 4.9. Statistical Analysis

GraphPad Prism 6 software was used to analyze the statistical difference among the treatment groups. Each data set was tested for normality using the Shapiro Wilk test for Normality. Normally distributed data sets were analyzed using a one-way Analysis of Variance (ANOVA) and non-parametric distributed data sets were analyzed using the Kruskal–Wallis test. Differences at a two-tailed *p*-value ≤ 0.05 were considered statistically significant. Parametric and non-parametric data sets with a statistically significant *p*-value were then subjected to Tukey’s posthoc test or Dunn’s Multiple comparison tests, respectively, to compare differences between each treatment group. The data shown represent the mean ± standard error of the mean (SEM) for the biological replicates and statistical differences were determined at a two-tailed *p*-value ≤ 0.05, therefore, any differences with *p* ≤ 0.05 were considered significant.

## 5. Conclusions

In conclusion, this study optimizes miR-21 inhibition using LNA technology in bovine granulosa cells, confirms the LNA specificity, and validates a functional knockdown of this crucial posttranscriptional regulator. The antiapoptotic role of miR-21 was confirmed with miR-21 directly regulating PDCD4, but not PTEN at the protein, but not at the transcript level. This study also suggests miRNA-miRNA interaction with altered miR-155 levels in miR-21 inhibited cells. miR-21 is the most abundant miRNA in bovine granulosa cells and is also reported to be the most affected by EDCs such as BPA [100]. This study shows BPA-mediated elevation in miR-21 expression around 12 h post-exposure. One important consideration is the antiapoptotic and proapoptotic functions of miR-21 and BPA, respectively, with both miR-21 and apoptosis found to be increased [26,27,101,102]. This may seem like a counterproductive finding, but heavily implies that BPA may induce apoptosis through a pathway that does not involve miR-21 signaling. In contrast under certain conditions, BPA may act through miR-21 and PDCD4 to induce antiapoptotic prooncogenic effects in cells. This explains a potential mechanism by which BPA induces cancerous malignancies. This needs to be confirmed by future research that expands on apoptotic mechanisms. The general consensus has suggested that the influence of BPA on individual miRNA transcription is largely owing to ERE upstream of specific miRNA promoters [67]. This study confirms this by showing BPA-mediated increases in the expression of two upstream miR-21 regulators, VMP1 and STAT3. The downstream changes in PDCD4 protein levels after miR-21 LNA transfections and BPA treatment suggests a new plausible mechanism for BPA toxicity in bovine GCs that is not unlike those seen in various cancers. BPA induces epigenetic alterations in the female reproductive tract and its mechanism of action on miRNAs should be subject to extensive research. Identifying these additional pathways will enhance our understanding of the roles individual miRNAs play in normal versus abnormal ovarian biology and fertility under environmental chemical exposure.

## Figures and Tables

**Table 1:** MicroRNA primers for qPCR.

MicroRNA	Primer ID	Accession Number	Sequence (5′-3′)	E (%)	Source
miR-191	hsa-miR-191-5p	MIMAT0000440	AACGGAATCCCAAAAGCAG	99.7	[88]
miR-106a	hsa-miR-106a-5p	MIMAT0000103	CGCCAAAAGTGCTTACAGTGC	92.4	
miR-21	bta-miR-21-5p	MIMAT0003528	TAGCTTATCAGACTGATGTTGACT	96.7	[89]
miR-34c	bta-miR-34c	MIMAT0003854	AGGCAGTGTAGTTAGCTGATTGC	99.6	[90]
miR-155	hsa-miR-155-5p	MIMAT0000646	TGCTAATCGTGATAGGGGTAAA	100	[91]
miR-146a	bta-miR-146a	MIMAT0009236	TGAGAACTGAATTCCATAGGTTG	100.2	[92]
miR-10b	hsa-miR-10b-3p	MIMAT0000267	GACAGATTCGATTCTAGGGGAAT	101.5	[93]

**Table 2:** mRNA primers for qPCR.

Gene Symbol	Gene Name	Product Size (bp)	Accession Number	Primer Sequence Sets (5′-3′)	E (%)	Source
YWHAZ	*Tyrosine 3-monooxygenase/tryptophan 5-monooxygenase activation protein zeta*	120	NM_174814.2	F: GCATCCCACAGACTATTTCC R: GCAAAGACAATGACAGACCA	100.3	[94]
ACTB	*Beta-actin*	186	NM_173979.3	F: CCTTCCTGGGCATGGAATCCT R: TCTTCATTGTGCTGGGTGCC	97	[15]
GAPDH	*Glyceraldehyde-3-phosphate dehydrogenase*	153	NM_001034034.2	F: TTCCTGGTACGACAATGAATTTG R: GGAGATGGGGCAGGACTC	99.8	[95]
PDCD4	*Programmed cell death 4*	108	NM_001083647.1	F: AAAGACTCTGACACCGATTA R: CAAGGACACTGCCAACAC	99.4	[96]
PTEN	*Phosphatase and tensin homolog*	151	NM_001319898.1	F: TGCAGAGTTGCACAGTATCCC R: CACCAGTTCGTCCCTTTCCA	100	[97]
VMP1	*Vacuole membrane protein 1*	266	NM_001075368.2	F: GACCAGAGACGTGTAGCAATG R: ACAATGCTTTGACGATGCCATA	99.4	[72]
STAT3	*Signal transducer and activator of transcription 3*	200	NM_001012671.2	F: GTGCATTGACAAAGACTCCG R: AATCAGGGAGGCATCACAAT	100.1	[98]

## Data Availability

Not applicable.

## Supplementary Materials

The following supporting information can be downloaded at: https://www.mdpi.com/article/10.3390/ijms23158276/s1.
