# Supplementary material for: BPA Decreases PDCD4 in Bovine Granulosa Cells Independently of miR-21 Inhibition
Source: Int J Mol Sci. 2022 Jul 27;23(15):8276. doi: 10.3390/ijms23158276 (PMC9368835; doi:10.3390/ijms23158276)
Supplement: Supplementary file 1 [file ijms-23-08276-s001.zip › ijms-1784737-supplementary.pdf]

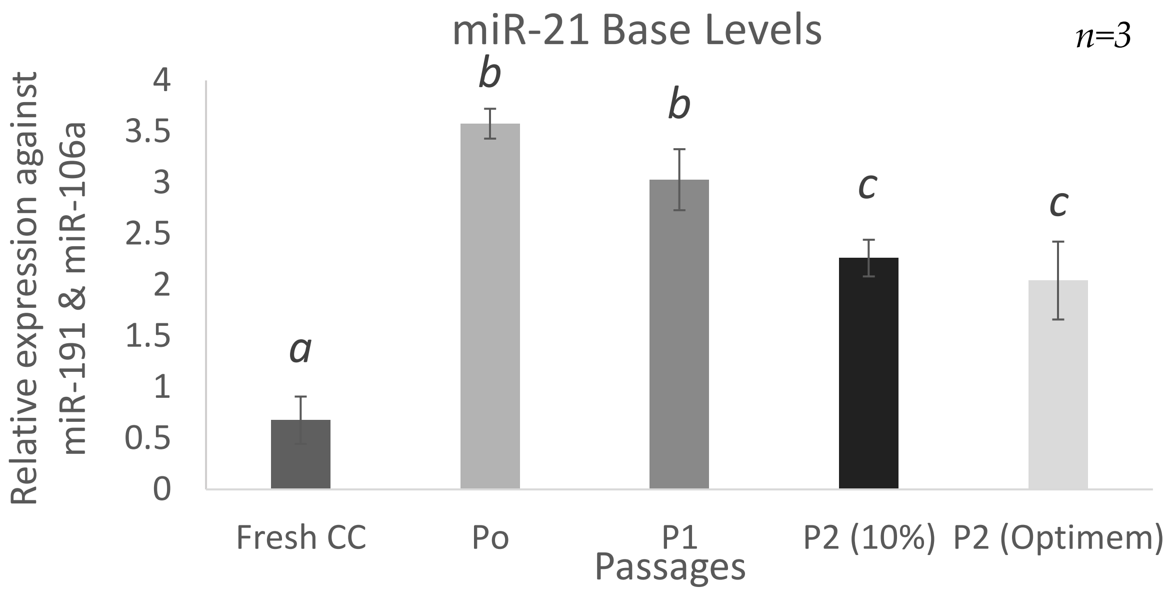

Supplementary Figure S1. mi-21 levels upon plating, throughout passaging, and after serum restriction.

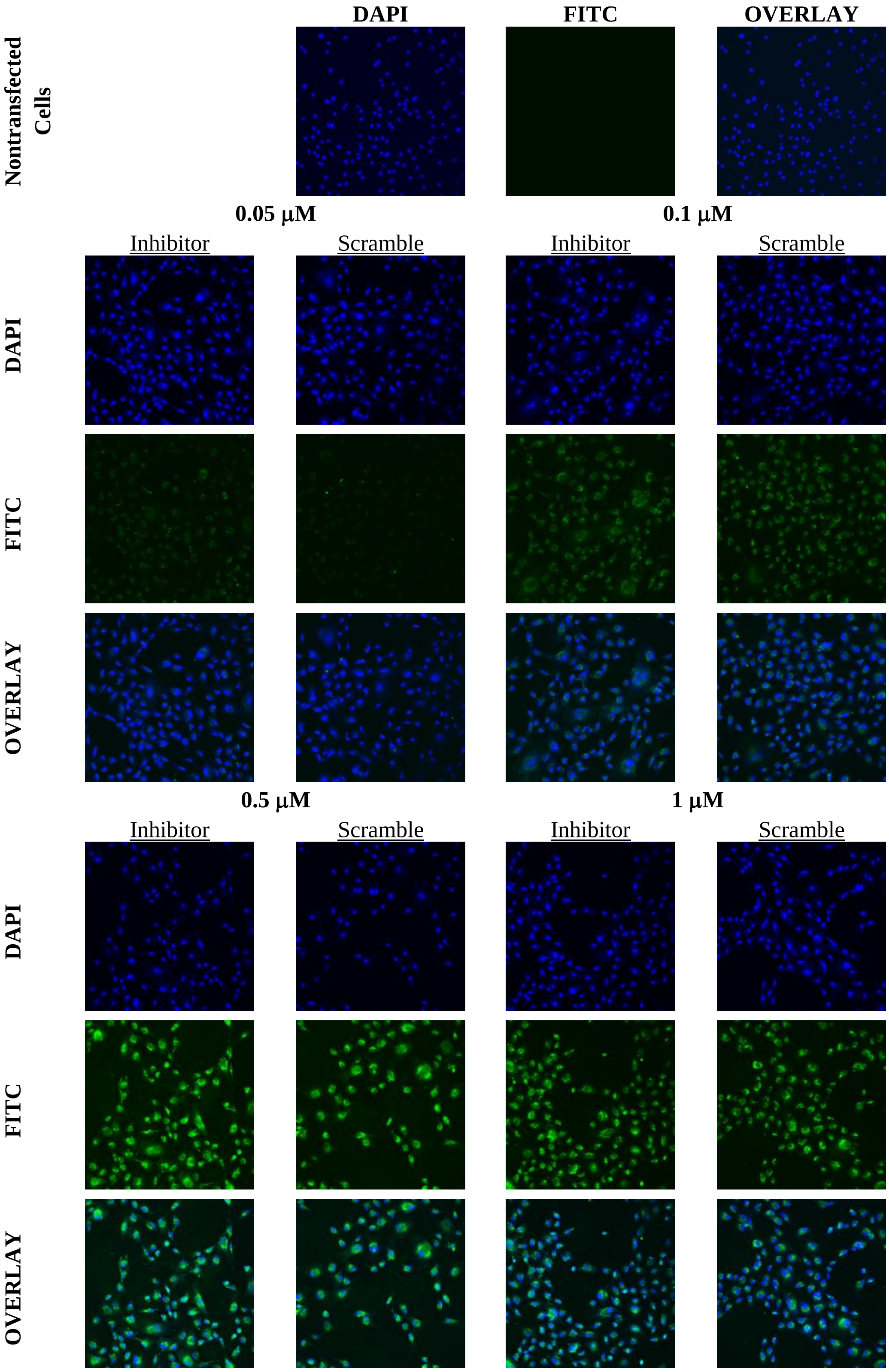
DAPI
FITC
OVERLAY
Nontransfected Cells
0.05 μM
0.1 μM
Inhibitor
Scramble
Inhibitor
Scramble
DAPI
FITC
OVERLAY
0.5 μM
1 μM
Inhibitor
Scramble
Inhibitor
Scramble
DAPI
FITC
OVERLAY

(A)

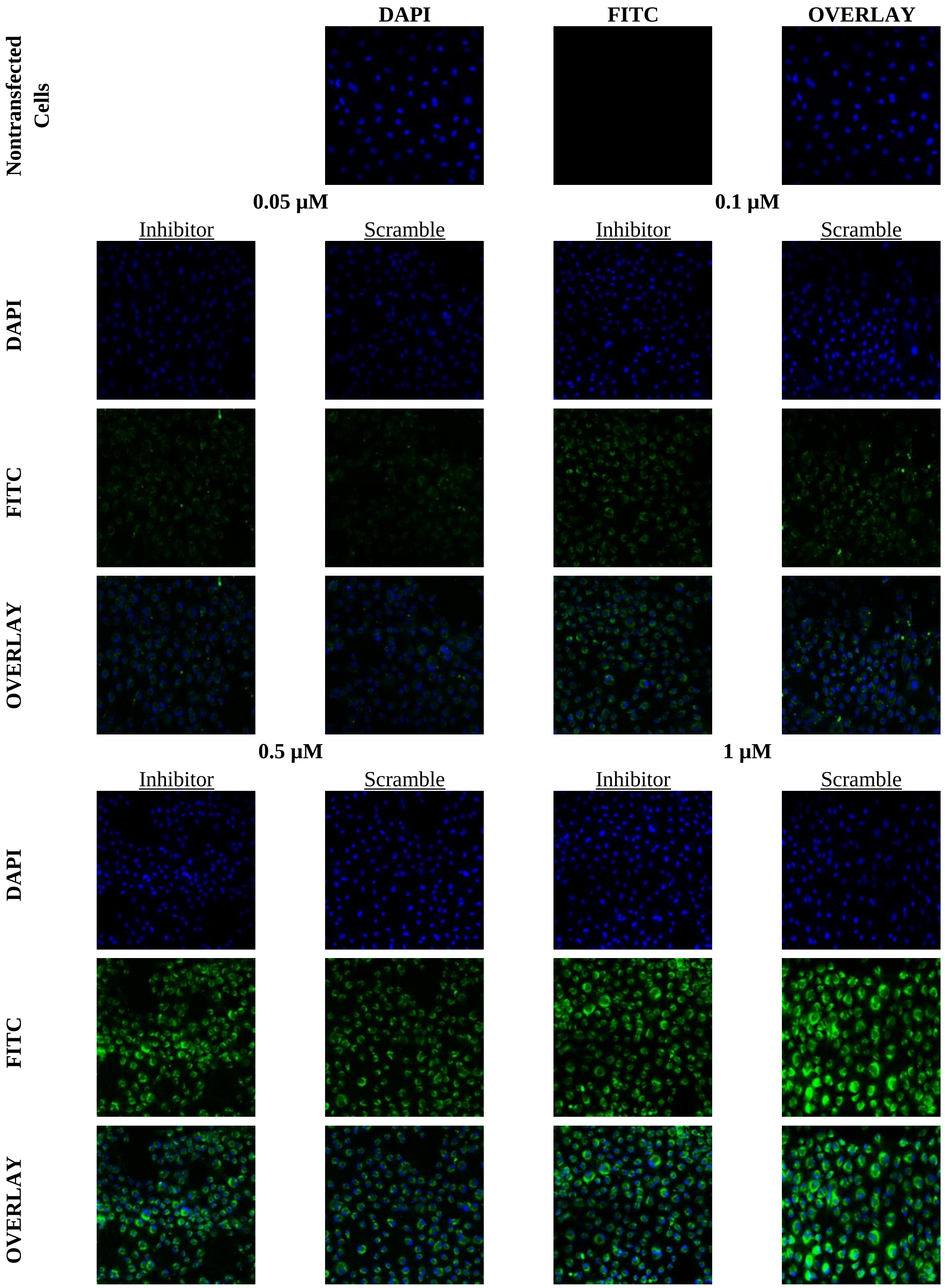
DAPI
FITC
OVERLAY
Nontransfected Cells
0.05 μM
0.1 μM
Inhibitor
Scramble
Inhibitor
Scramble
DAPI
FITC
OVERLAY
0.5 μM
1 μM
Inhibitor
Scramble
Inhibitor
Scramble
DAPI
FITC
OVERLAY

(B)

(C)

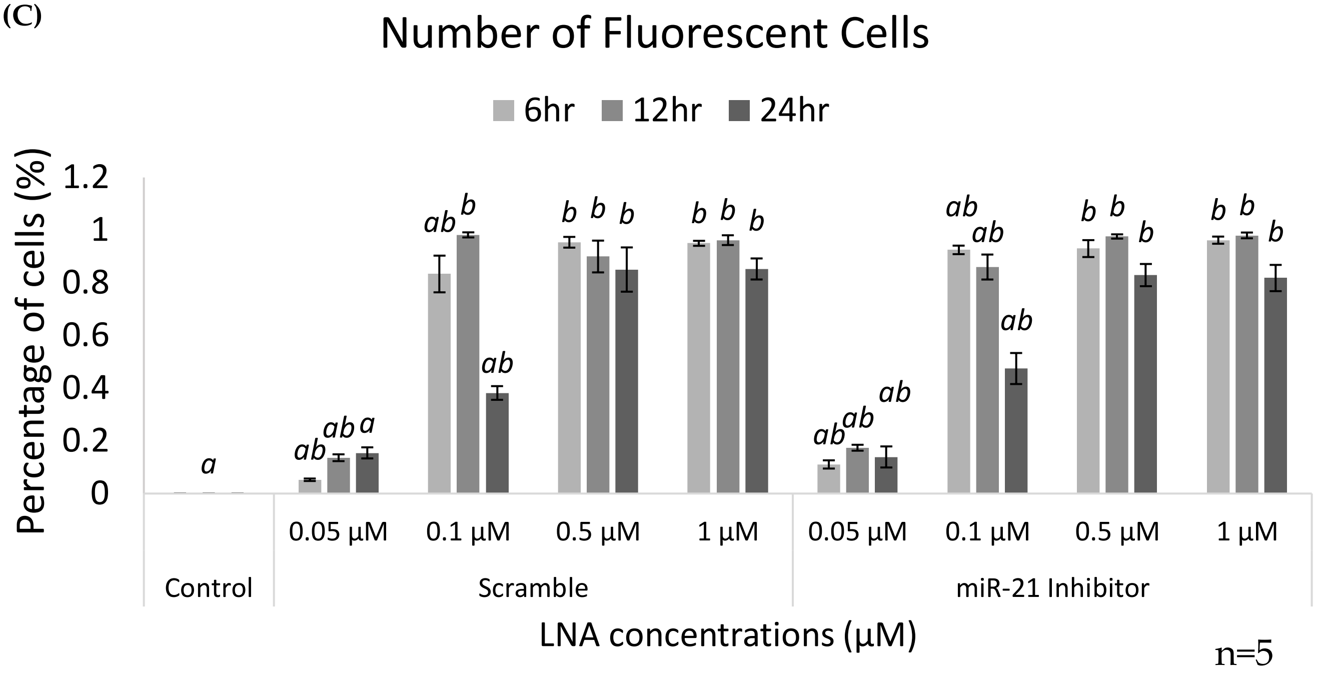

Supplementary Figure S2. Additional Confocal microscopy of LNAs at (A) 6 and (B) 24hrs with (C) number of fluorescent cells

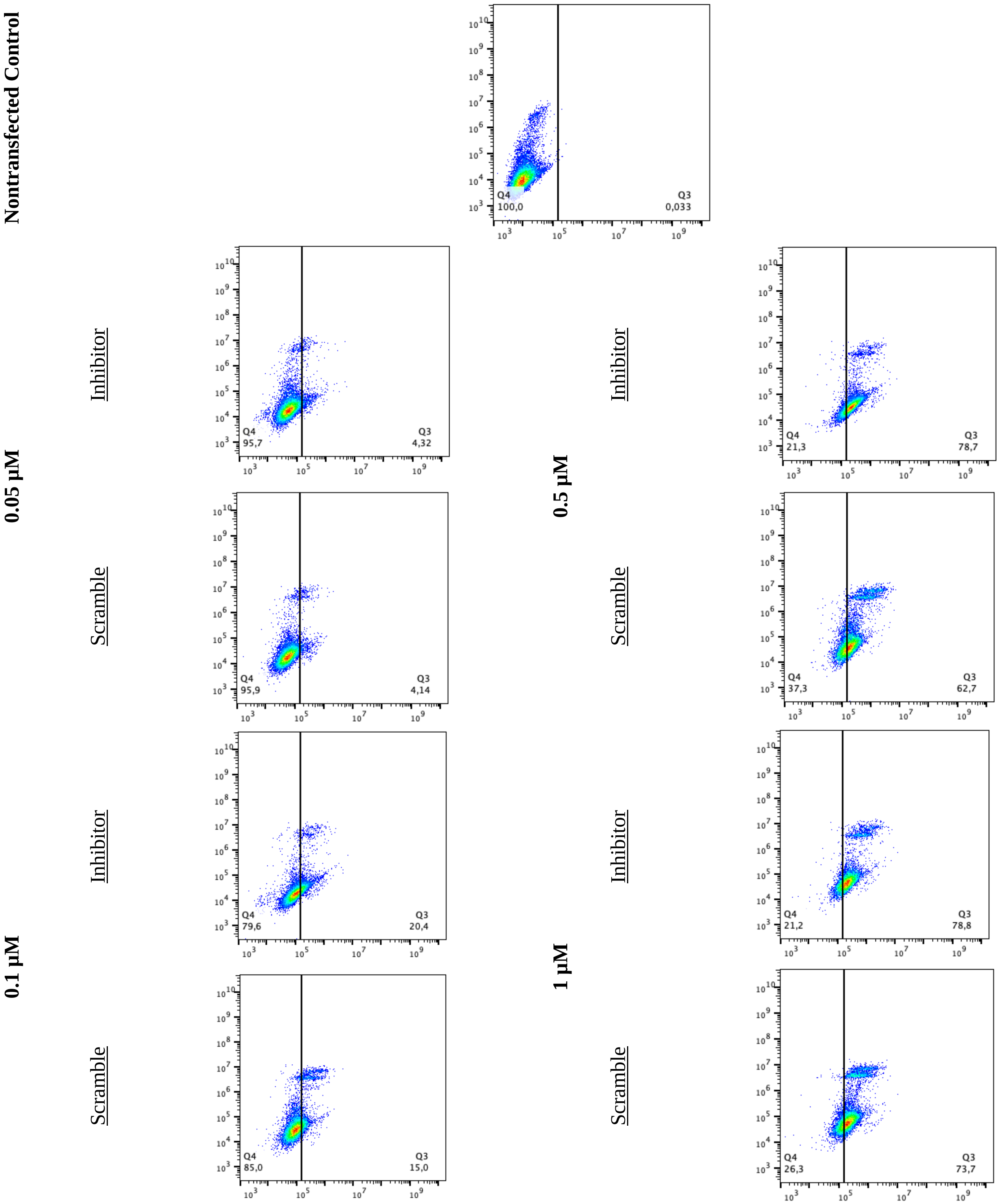
Nontransfected Control
Q4
100,0
Q3
0,033
0.05 μM
Inhibitor
Q4
95,7
Q3
4,32
Scramble
Q4
95,9
Q3
4,14
0.5 μM
Inhibitor
Q4
21,3
Q3
78,7
Scramble
Q4
37,3
Q3
62,7
0.1 μM
Inhibitor
Q4
79,6
Q3
20,4
Scramble
Q4
85,0
Q3
15,0
1 μM
Inhibitor
Q4
21,2
Q3
78,8
Scramble
Q4
26,3
Q3
73,7

(A)

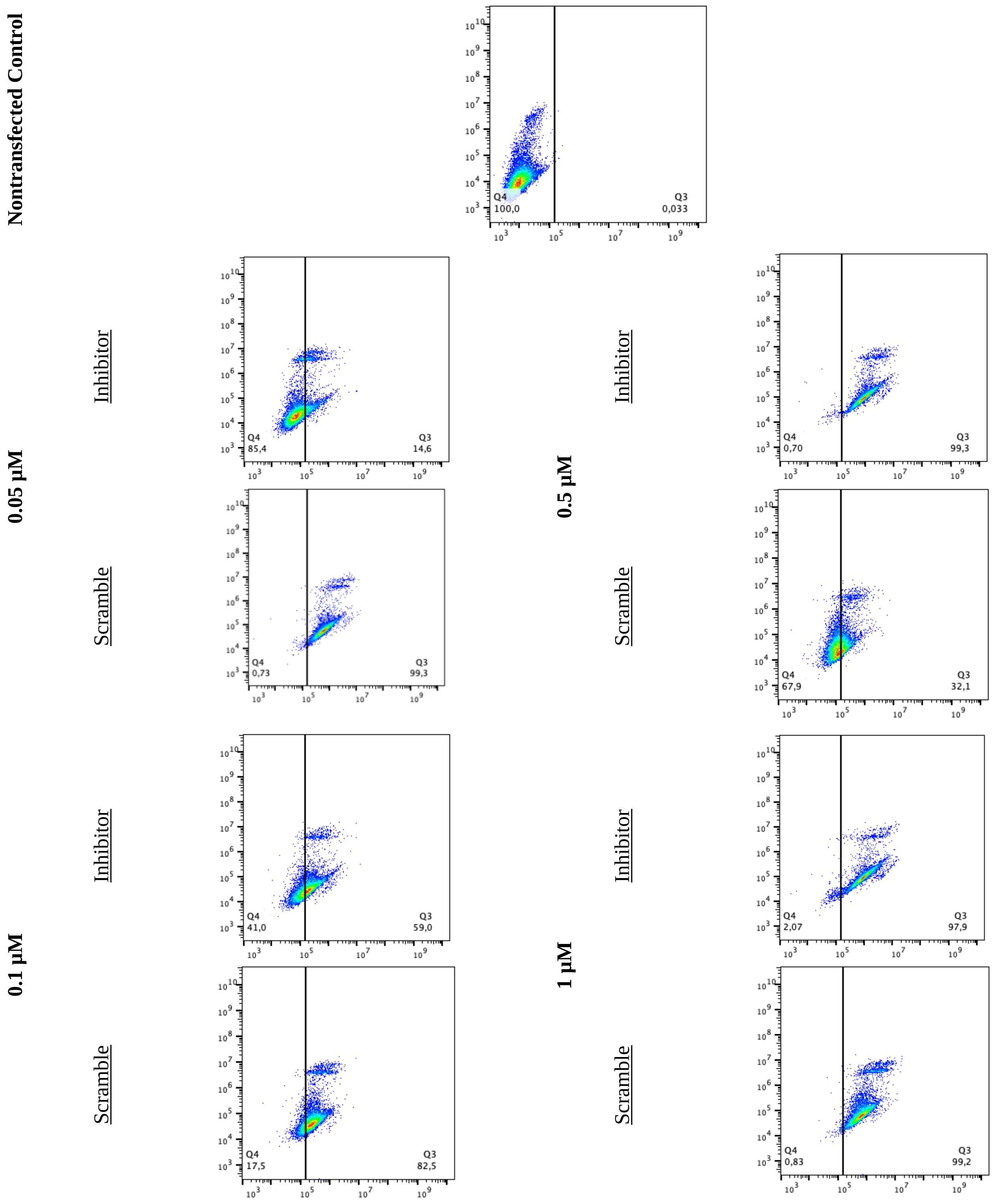

(B)

Supplementary Figure S3. Additional Flow Cytometry of LNAs at (A) 6 and (B) 24hrs.

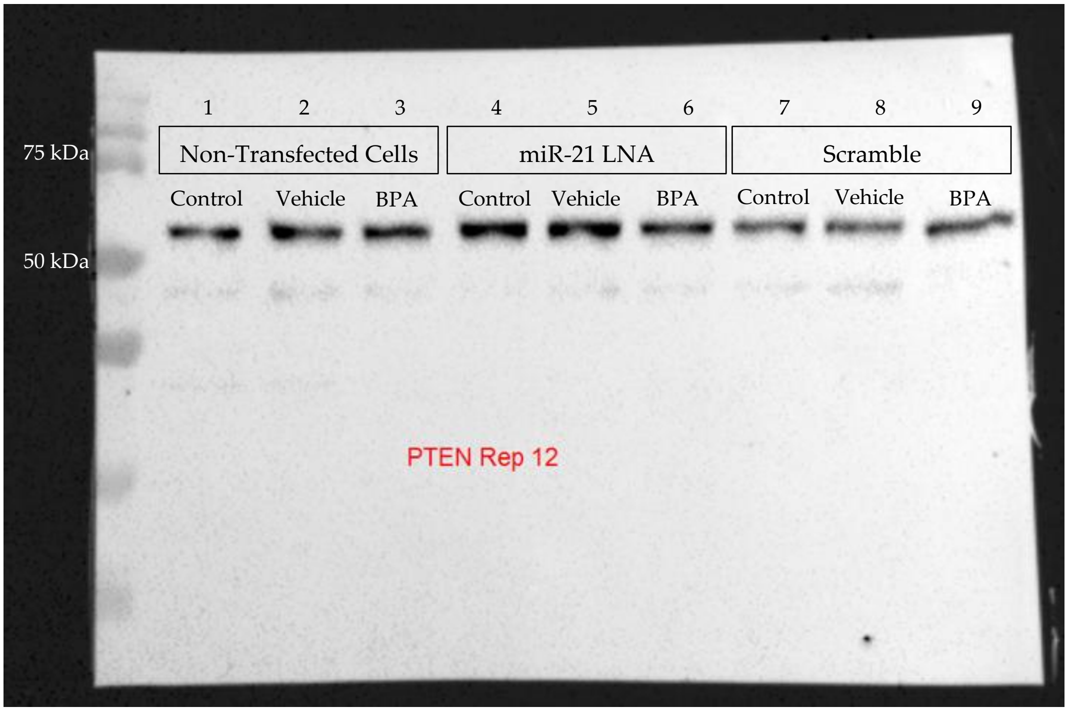

Supplementary Figure S4. Original PTEN blot after miR-21 LNA and BPA treatment.

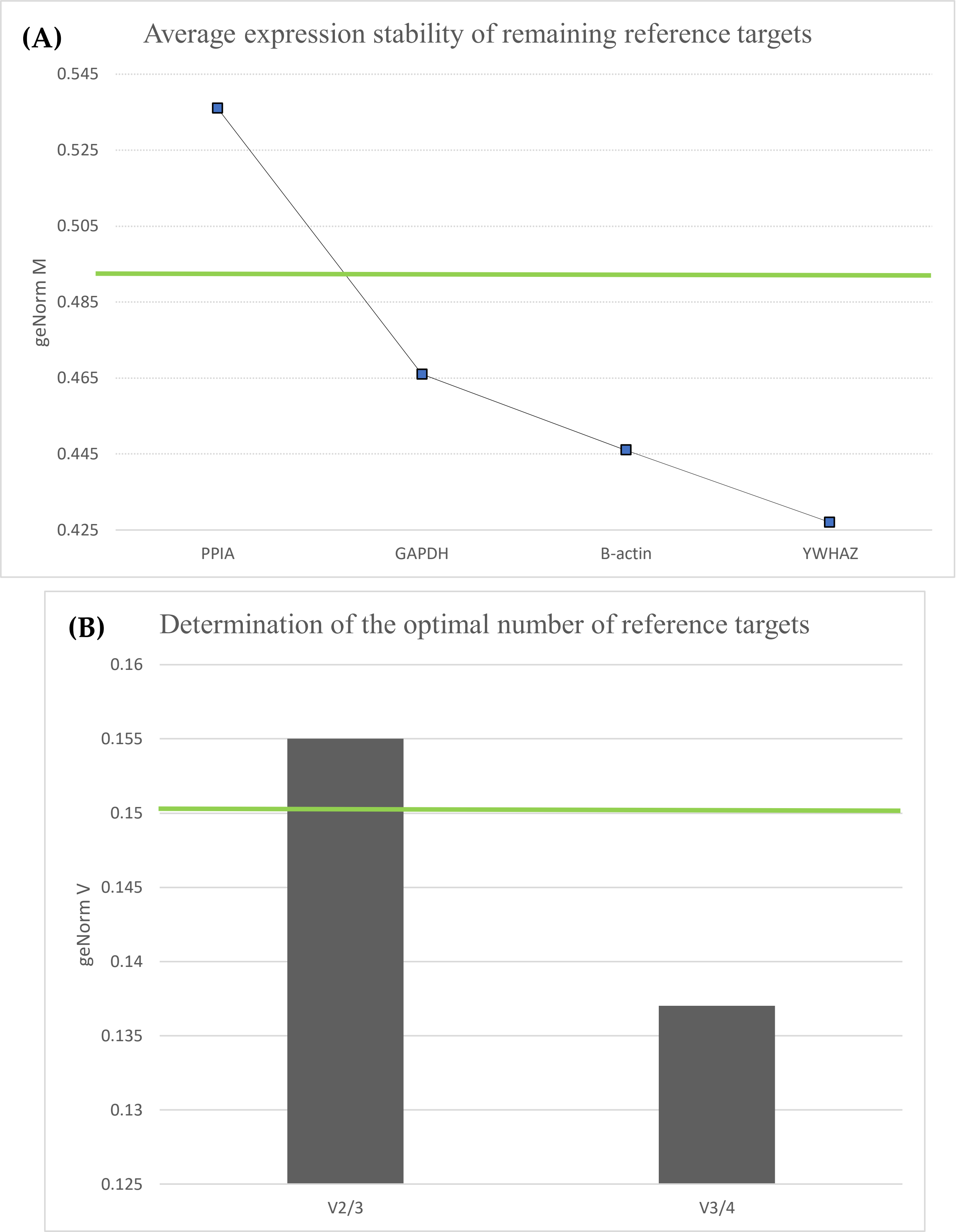

Supplementary Figure S5. **Optimal reference gene selection (A) and number of references (B) across treatments using geNorm.**
